# Long-term and persistent vocal plasticity in adult bats

**DOI:** 10.1038/s41467-019-11350-2

**Published:** 2019-07-29

**Authors:** Daria Genzel, Janki Desai, Elana Paras, Michael M. Yartsev

**Affiliations:** 1 Helen Wills Neuroscience Institute and Department of Bioengineering, UC Berkeley, Berkeley, CA 94720 USA; 2 Department of Integrative Biology, UC Berkeley, Berkeley, CA 94720 USA; 3 Department of Environmental Science, Policy, and Management, UC Berkeley, Berkeley, CA 94720 USA

**Keywords:** Neuroscience, Learning and memory, Animal behaviour, Social behaviour

## Abstract

Bats exhibit a diverse and complex vocabulary of social communication calls some of which are believed to be learned during development. This ability to produce learned, species-specific vocalizations – a rare trait in the animal kingdom – requires a high-degree of vocal plasticity. Bats live extremely long lives in highly complex and dynamic social environments, which suggests that they might also retain a high degree of vocal plasticity in adulthood, much as humans do. Here, we report persistent vocal plasticity in adult bats (*Rousettus aegyptiacus*) following exposure to broad-band, acoustic perturbation. Our results show that adult bats can not only modify distinct parameters of their vocalizations, but that these changes persist even after noise cessation – in some cases lasting several weeks or months. Combined, these findings underscore the potential importance of bats as a model organism for studies of vocal plasticity, including in adulthood.

## Abstract (web summary)

Bats are long-lived animals that can produce a complex vocabulary of social communication calls. Here, the authors show that even in adulthood, bats retain the ability to adaptively introduce long-term modifications to their vocalizations, showing persistent vocal plasticity.

## Introduction

Humans are renowned for the ability to learn their vocalizations and maintain vocal plasticity throughout life. This ability allows them to modify and adjust properties of their vocal repertoire in response to auditory inputs from their surroundings^1–3^. Amongst animals (particularly mammals) very few species share this capacity^4–11^ resulting in a sparsity of relevant model organisms for neurobiological studies of this trait. To date, nearly all detailed neurobiological investigations have been conducted in birds, with most focusing on plasticity in juveniles^6,12^ (but see Tumer and Brainard^13^). These studies have yielded invaluable insight and revealed many of the computations that might support the bird’s capacity of song imitation as well as its potential parallel to human speech^6^. At the same time, the field has also faced translational challenges due to evolutionary divergence between avian and mammalian brains (e.g. the nuclear organization of the avian pallium vs. the six-layered organization of mammalian cortex)^14^. Thus, the absence of a tractable mammalian model system for studying mechanisms of vocal plasticity, particularly in adulthood, represents a major and long-standing gap in the field.

Recently, bats have been proposed as a potential mammalian model system for bridging this gap owing to their unique specialization in acoustic signaling^15,16^. In addition to their extraordinary echolocation capability, bats also possess a rich and diverse repertoire of social communication calls that are used primarily (if not exclusively) for social interactions^15–18^. Importantly, some of the bats’ social vocalizations are believed to be learned during development by attending to auditory signaling produced by conspecifics in their surroundings. This rare ability is posited to be an extreme form of long-term and persistent vocal plasticity in a developing mammal^8,9,17–19^.

To what extent is bat vocal plasticity retained in adulthood? The ethology of a bat’s life suggests that maintaining the capacity for vocal plasticity beyond development could be extremely advantageous. Bats are amongst the longest living mammals (comparing lifespan relative to body size^20^) and spend much of their lives in socially complex and highly dynamic environments where effective acoustic interactions need to occur under noisy conditions. For example, Egyptian fruit bats (*Rousettus aegyptiacus)* live in large cave dwellings with many thousands of individuals^21^ where ambient noise levels can reach up to 100 dB SPL (Y. Yovel, personal communication). Under such varying and complex acoustic conditions, bats would clearly benefit from retaining a high degree of vocal plasticity as part of an adaptive, robust and effective vocal communication system. This notion, however, remains largely unexplored, especially in response to structured acoustic perturbation under controlled laboratory conditions (but see Boughman et al.^18^).

Here, we set out to examine the extent of vocal plasticity in adult Egyptian fruit bats for two major reasons. First, recent studies have demonstrated the crucial role of auditory feedback during development in shaping their social vocalizations, underscoring the potential validity of this species as a mammalian model organism for vocal plasticity and learning^8,19^. Second, a suite of novel methodologies has been developed and customized for adult Egyptian fruit bats, enabling neurophysiological measurements during uninhibited social interactions^22^ and even during free flight^23,24^. Yet, two major questions must first be addressed in order to support the use of adult Egyptian fruit bats for studies of vocal plasticity under laboratory conditions. First, does this species retain a capacity for vocal plasticity in adulthood, and if so, can it be engaged and studied under laboratory conditions? Addressing these two major questions was the goal of the present study where we leveraged the natural behavior of Egyptian fruit bats to vocally communicate even under acoustically noisy conditions.

**Fig. 1:**
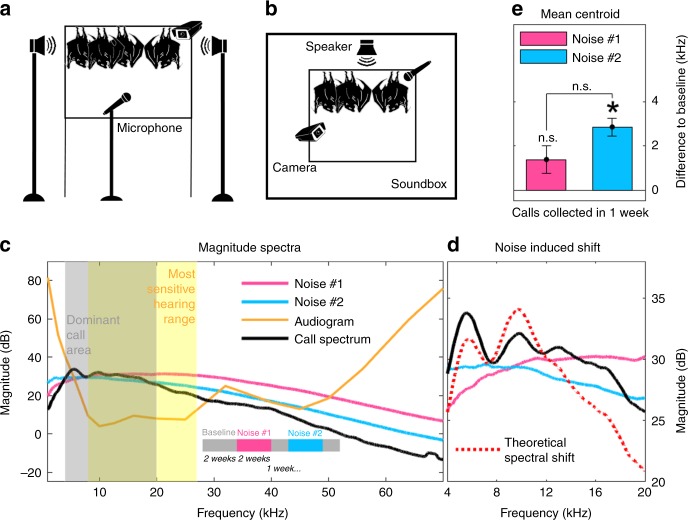
Experimental setup and hypothesis. **a**, **b** illustration of the experimental setup with microphones, speakers and camera positions depicted for Groups 1 and 2 (**a**) and for Groups 3 and 4 (**b**). **c** magnitude spectra for the first and second noise condition, the audiogram for *R. aegyptiacus* (adapted from^30^) and the typical social calls’ spectrum of *R. aegyptiacus*. The timeline for silence and noise presentation is visualized in the lower part of the graph. The hypothesis is that when confronted with noise in their most sensitive hearing range (indicated by the yellow shaded area and the orange line representing their audiogram^30^) or in the dominant frequency area of their call (indicated by the gray shaded area), bats would shift the energy of their calls into higher or lower frequency areas to escape the noise and optimize their signal-to-noise ratio. The first noise (noise #1, in pink, with more energy in higher frequency bands) could result in a downward or upward shift, the second noise (noise #2, in blue, with more energy in the low to mid frequency range) should result in an upward shift as the higher frequency areas are less contaminated by the noise. **d** shows a detailed view of the calls’ most dominant frequency area and a theoretical upward shift and redistribution of the calls’ energy in relationship to the spectra of the noise. By focusing more energy in a higher frequency band, the overall frequency bandwidth might be decreased. **e** mean call centroid and corrected standard error (over all summarized calls collected in the week of silence) for Groups 1 and 2 after noise #1 and noise #2 are plotted in terms of their difference to the mean baseline. Notations above each bar indicate either significance (*α* = 0.0045, t-test through mixed linear model) relative to baseline or the other noise condition with an asterisk or not significant with n.s

Acoustic noise can mask communication signals, thereby disrupting information transfer and leading to errors in detection or interpretation of the signals by the receiver^25–27^. It has been shown that most animals—from insects, amphibians, and birds to mammals—modify their acoustic signals (amplitude, duration and spectral composition) to maintain the signal-to-noise ratio of their vocalizations in the presence of noise^28,29^, thereby increasing the probability of successful communication^27^. Therefore, we designed an ethological behavioral paradigm to investigate how bats adapt the acoustic parameters of their vocalizations to the spectral features of their surroundings under controlled laboratory conditions (Fig. 1). Importantly, the focus of this study was to specifically assess whether these adaptive changes would persist after cessation of the acoustic perturbation (i.e., in the complete absence of noise), which suggests long-term and persistent vocal plasticity.

In its social environment, the Egyptian fruit bat is a highly vocal bat, and its hearing sensitivity and call frequency distribution are well characterized^17,30,31^(Fig. 1c). By specifically tailoring spectral shapes of a continuous, broad-band background noise, we were able to target the bats’ most sensitive hearing range or the most dominant call frequency range of their social calls (see Methods). We hypothesized that the bats’ communication calls should exhibit changes in their spectral features due to the influences of the presented acoustic perturbation. Figure 1d depicts a theoretical shift of call frequency correlated with spectral properties of the noise, where the mean magnitude spectrum of the bats’ calls before and after a theoretical shift is depicted in black and red (dashed line), respectively. When exposed to a noise with more energy in distinct frequencies, the bats can acoustically “escape” into lower or higher frequency bands to optimize their signal-to-noise ratio. Since the background noise was presented to the bats continuously (requiring them to communicate acoustically in noisier conditions), we hypothesized that the observed frequency shift in the calls might persist even after the noise was silenced—indicative of vocal plasticity. Hence, after noise cessation, we continuously monitored all social communication calls occurring during the bats’ natural interactions to assess the long-term effect of the acoustic interference.

We find that after noise cessation, adult bats modify distinct parameters of their vocalizations to minimize interference with the acoustic perturbation, and the observed shifts persist for up to several weeks or months after noise cessation. The obtained results demonstrate that this species retains the capacity for vocal plasticity in adulthood, underscoring its potential importance as a model organism for studies of vocal plasticity throughout stages of development.

## Results

### Vocal plasticity in response to acoustic perturbation

Bats rarely call in isolation and interact in very close proximity to each other such that individual identification of who is calling is unreliable. We thus recorded from six independent groups of bats. Two of the six groups exhibited a very low call rate of <80 calls per day during the baseline period prior to noise manipulation and were therefore excluded from the experiment and not subjected to the noise perturbation. The nature of vocal interaction in this species of bats is pairwise, and in agreement with previous reports^31^ we did not observe individual bats dominating the ‘conversation’. We most often observed multiple differently composed groups of bats engaging in vocal interactions, as reported previously (ref. ^31^).

We initially examined several distinct noise conditions: the first noise condition (noise #1, pink curve, Fig. 1c and Supplementary Fig. 1a) spanned much of the bats’ most sensitive hearing range and call spectrum (8–27 kHz). The second noise condition (noise #2, blue curve, Fig. 1c and Supplementary Fig. 1a), displayed a steeper slope and had a higher magnitude in the more dominant frequencies of the Egyptian fruit bat call spectrum (4–20 kHz). A third noise condition (noise #3, presented only to Group 1, Supplementary Fig. 1A) had the steepest spectral slope and energy mainly in the low frequency range (below 5 kHz). The last noise condition was only tested in one group and hence not included in the main analysis but is reported in Supplementary Fig. 1. Before noise presentation, all groups were acclimated for one week to the housing environment in which the acoustic perturbation took place. After 2 additional weeks of baseline recording, noise perturbation commenced. The acoustic perturbation consisted of low amplitude (45–50 dB, as used in prior studies in bats^32,33^), continuous presentation of background noise over a period of 2 weeks. This amplitude level was chosen in order to elicit changes in vocalizations without disturbing the bats such that they would cease their vocal interactions. In total, we recorded 6348, 17115, 14153, and 10256 calls during the baseline period for Groups 1, 2, 3, and 4, respectively. The critical measurements of the experiment occurred following the cessation of the acoustic perturbation periods where we recorded the bats vocalizations for at least one additional week in order to assess the influence of noise perturbation on their vocalizations. All recorded vocalizations were social communication calls occurring during the bats’ natural interactions (see examples in Supplementary Fig. 2).

The first two groups in the experiment consisted of six bats each that were recorded in a large enclosure (Fig. 1a; see Methods). The background noise was designed to contain more energy in the most sensitive hearing range (8–27 kHz) of the adult bat and tapered off toward the lower and higher frequencies (Fig. 1c and Supplementary Fig. 1A). We hypothesized that adult bats of this species might adapt their vocalizations to minimize interference with the presented noise perturbation. Furthermore, if adult bats do retain the capacity for vocal plasticity in adulthood, then this vocal adaptation should persist after cessation of the acoustic perturbation. Previous studies^32,33^ have shown that bats shift the centroid of their echolocation calls (amongst other call parameters) during noise presentation contingent upon background noise. Thus, initial examination of our hypothesis focused on the spectral centroid (the calls’ spectral center of mass)^34^ of the bats’ social vocalizations by comparing this measure before (baseline) and after presentation of each of the noise conditions. We hypothesized that any changes in the centroid should not result in strong reorganizations of the call structure, since only the energy distribution of the call is shifted within its own frequency range, allowing escape from the masking noise (see Fig. 1d).

During the week of silence after the presentation of noise #1 (pink curve in Fig. 1c) we recorded a total of 3442 calls for Group 1 and 10770 calls for Group 2. A summarization procedure into observation bins was applied on all calls (see Methods) and all further analysis was done on the summarized data sets. These summarized calls were compared to all summarized calls recorded during baseline. We found that not only did the bats adapt their vocalizations by shifting their center of mass upwards but that this adaptation persisted in the complete absence of the noise (although this change was not significantly different from baseline when Bonferroni corrected for 11 multiple comparisons, see Methods; Fig. 1e, 1.39 ± 0.623 kHz for both groups, *t*(86.6) = 2.23 and *p* = 0.028, *n* = 537 bins, *t*-test through mixed linear model, see Methods). This finding was also not significant for both groups individually (Supplementary Fig. 1B,C; 0.471 ± 0.75 kHz, *z* = 0.628 and *p* = 0.53, *n* = 139 bins for Group 1, 1.69 ± 0.811 kHz, *z* = 2.083 and *p* = 0.037, *n* = 398 bins for Group 2, *z*-test through AR(1) model, see Methods).

The observed, moderate shift suggests that adult bats may retain a form of long-term plasticity for vocal production, which allows them to change the acoustic features of their vocalizations in response to changes in the acoustics of their environment. To further explore this hypothesis, we next examined whether the observed persistent shift depends on the spectral properties of the presented acoustic perturbation. To assess this possibility, both groups were subsequently exposed to noise #2, which differed in having a steeper spectral slope (blue curve in Fig. 1c and Supplementary Fig. 1A). We reasoned that because a larger portion of the bats’ most dominant call frequencies would now be perturbed, this manipulation would further drive the bats to increase their spectral center of mass to avoid the noise. During the week of silence after the presentation of noise #2, we recorded 5704 calls for Group 1 and 18272 calls for Group 2. Indeed, (following the same summarization procedure) we found that the mean centroid of the calls recorded after the cessation of noise #2 was further shifted upward compared to baseline by 3.118 ± 0.545 kHz for Group 1 and 2.795 ± 0.515 kHz for Group 2 (*z* = 5.726 and *p* < 0.001, *n* = 171 bins for Group 1, *z* = 5.424 and *p* < 0.001, *n* = 505 bins for Group 2, *z*-test through AR(1) model), as well as, when compared to noise #1 by 2.627 ± 0.761 kHz for Group 1 and 1.091 ± 0.633 kHz for Group 2 (*z* = 3.452 and *p* < 0.001, *n* = 130 bins for Group 1, *n* = 415 bins, *z* = 1.723 and *p* = 0.085 for Group 2, z-test through AR(1) model). The mean shift for both groups was 2.87 ± 0.404 kHz relative to baseline (Fig. 1e, *t*(144) = 7.11 and *p* < 0.001, *n* = 676 bins, *t*-test through mixed linear model). The difference between the mean shifts for both groups from both noise conditions was 1.463 ± 0.511 kHz (*t*(107) = 2.86 and *p* = 0.0051, *n* = 545 bins, *t*-test through mixed linear model). Single group data is depicted in Supplementary Fig. 1B, C. One of the groups (Group 1) was further exposed to background noise with an even higher slope (noise #3, green curve in Supplementary Fig. 1A), but did not exhibit a further shift upwards (see Supplementary Fig. 1B). Hence, we focused on noise #2 for all subsequent testing.

### Persistence of spectral centroid shift

To further assess the robustness of the observed effect, we repeated the experiment in two additional groups of bats (Groups 3 and 4). These two groups were recorded inside acoustic sound-chambers (40–70 dB attenuation) to better control for environmental conditions (Fig. 1b). During the week of silence after noise presentation, we recorded 6314 calls for Group 3 and 3557 calls for Group 4. The calls were summarized and compared to all summarized calls recorded during baseline. Consistent with the results obtained from the first two groups of bats, we found again that the mean centroid of the calls shifted significantly upward relative to baseline (*z* = 7.389 and *p* < 0.001, 5.724 ± 0.775 kHz, *n* = 292 bins for Group 3, *z* = 3.484 and *p* < 0.001, 6.65 ± 1.909 kHz, *n* = 197 bins for Group 4, *z*-test through AR(1) model).

**Fig. 2:**
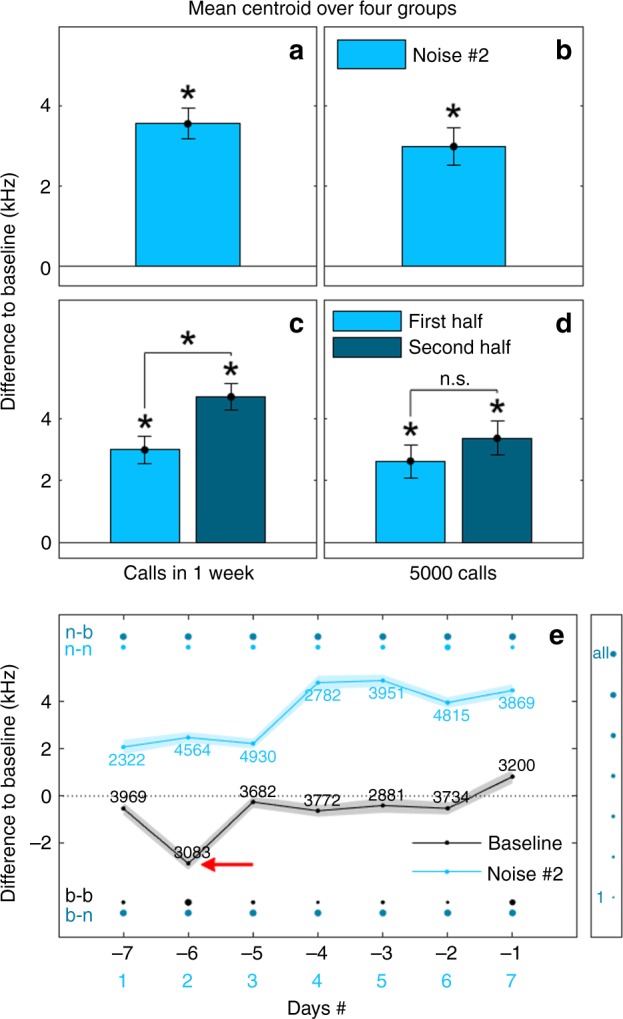
Centroid shift. **a**, **b** mean centroid shift and corrected standard error after noise cessation as compared to baseline across all four groups for the summarized 1-week data set (**a**) or the summarized 5000-data set (**b**). **c**, **d** mean centroid shifts and corrected standard errors for the first and second halves of the summarized 1-week data set (**c**) and for the first and seconds halves of the summarized 5000-data set (**d**). Notations above each bar indicate significance (*) or not (n.s.) when compared to baseline or the other data half, with *α* = 0.0045 and t-test through mixed linear model. **e** plotted is the mean difference in centroid compared to overall mean baseline (across all groups) on a day-by-day basis. The standard error is indicated by the shaded areas around each curve. Each data point is calculated by subtracting the mean value across all groups (for baseline or noise #2) from the overall mean baseline value for each day. Data is taken from the 1-week data sets (last week of baseline and first week of silence after noise #2). The row of dots ‘n-b’ depicts for each noise #2 data point how often it is significant to all baseline data points (range is from 0 —7 days). The row of dots (n-n) depicts for each day how often a noise #2 data point is significant to the other noise #2 data points (range is from 0 to 6 days). The same calculation is done for the baseline data points (b-n and b-b). The size of the dot scales with increasing number of significant days (see legend to the right: ‘all’ corresponds to 7 days for the n-b and b-n rows and to 6 days for the n-n and b-b rows; Wilcoxon rank sum test); e.g. n-b day 6 is significant to each baseline day (−7 to −1). It is important to note that, the centroid values only reach maximum significant days for the n-b and b-n comparisons except for the b-b data point marked by the red arrow. The numbers above each curve indicate how many calls were used to calculate each data point per day

In order to compare results across all groups, we looked at either 1) the same time window during which vocalizations occurred (same time of practice) or 2) the same number of vocalizations (same amount of practice) per group. Since calls for Groups 1 and 2 were recorded for a maximum timespan of 1 week after noise cessation, we looked at summarized calls collected during that 1-week period for all groups and compared these to summarized calls recorded during the last week of baseline (1-week data set, Fig. 2a). Similarly, the lowest number of calls recorded for any of the groups was ~5700 calls (Group 1, calls recorded during 1 week after noise cessation). We thus looked at 5000 calls for each condition and for each group (last 5000 baseline calls and the first 5000 calls after the noise perturbation, 5000-data set, Fig. 2b) and generated summarized data sets. All tested groups called consistently during the period after noise cessation (Supplementary Fig. 3). We found that the mean centroid of the calls across all groups, extracted for the summarized 1-week data set, as well as, for the summarized 5000-data set was significantly shifted relative to the bats’ baseline by 3.56 ± 0.383 kHz (*t*(175) = 9.3 and *p* < 0.001, *n* = 902 bins, t-test through mixed linear model) and 2.997 ± 0.468 kHz (*t*(108) = 6.4 and *p* < 0.001, *n* = 568 bins, t-test through mixed linear model), respectively, and plotted in Fig. 2a, b. Our results suggest that the observed effect was consistent for both time elapsed and amount of practice.

To analyze the stability of the observed centroid shift, we looked at the first and second half of the summarized data sets depicted in Fig. 2a, b. Calls were therefore divided based on their occurrence in the first or second half of the week following the acoustic perturbation or in the first or second half of the first 5000 calls recorded after the acoustic perturbation. This division allowed us to assess the potential decay of the effect as a function of time and amount of practice with the explicit prediction that if the effect is transient, then we would expect the shift to be smaller in the second half when compared to the first half. Interestingly, this was not the case. The mean centroid for the first and second half of the summarized 1-week data set shifted significantly relative to baseline by 2.983 ± 0.436 kHz and 4.689 ± 0.4102 kHz, respectively (*t*(177) = 6.84 and *p* < 0.001, *n* = 882 bins for the first half, *t*(186) = 11.43 and *p* < 0.001, *n* = 944 bins for the second half, *t*-test through mixed linear model) and is plotted in Fig. 2c. The mean centroid for the first and second half of the summarized 5000-data set shifted significantly relative to baseline by 2.625 ± 0.531 kHz and 3.358 ± 0.547 kHz, respectively (*t*(111) = 4.94 and *p* < 0.001, *n* = 600 bins for the first half, *t*(105) = 6.14 and *p* < 0.001, *n* = 600 bins for the second half, *t*-test through mixed linear model) and is plotted in Fig. 2d. All first and second half data sets were different from each other as well, but were only significant for the half week data sets (*t*(131) = 3.77 and *p* < 0.001, *n* = 654 bins for the two half 1-week data sets, *t*(82.9) = 1.3 and *p* = 0.198, *n* = 400 bins for the two half 5000-data sets, *t*-test through mixed linear model) and did not show a decay but, in fact, a further increase (the difference between the first and second half 1-week data set was 1.712 ± 0.454 and 0.738 ± 0.568 kHz between the first and second half 5000-data set).

To account for the natural variation of the groups’ social calls, we extracted mean centroid values on a day-by-day basis for each group and compared (across all groups) the difference in the daily value to all other days during baseline and post-noise perturbation periods (Fig. 2e). We found that the values were consistently different for all days between baseline and post-noise perturbation period. Our results thus far suggest that the low-amplitude acoustic perturbation induced a robust form of vocal plasticity across all group compositions and recording environments, which persisted for at least one week.

**Fig. 3:**
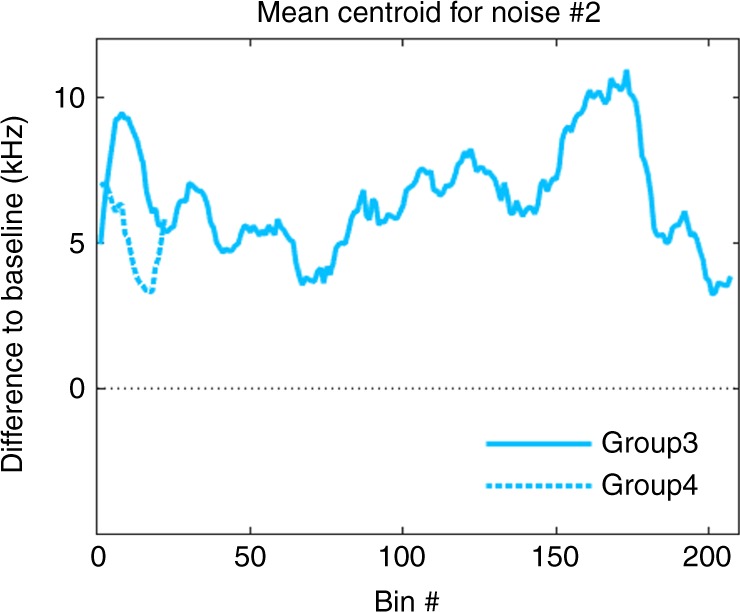
Stability of centroid shift over weeks and months. Groups 3 and 4 were recorded for an extended period of time after noise cessation (11 and 5 weeks, respectively) to assess the stability of the observed shift in centroid. Depicted is the development of the mean centroid in terms of difference from baseline. A bin number is generated by calculating the mean over 5000 calls and a sliding window of 500 calls. The low call rate for Group 4 resulted in the low bin number for this group. The mean centroid fluctuates for both groups but never returns to zero ( = baseline)

We further assessed whether the observed changes could be attributed to the fact that these signals were measured during entirely uninhibited, natural interactions which are inherently variable and complex. To do so, we compared the fluctuations of the centroid over a 1-week period during baseline and after-noise exposure for each group (Supplementary Fig. 4). This comparison revealed a clear and persistent separation between the baseline and after-noise curves which never overlapped for any group nor at any time point thus verifying that the observed shift in centroid is not due to random noise in the calls but is an effect of the noise perturbation. In addition, we continued recording Groups 3 and 4 for an extended period of time (5–11 weeks) after the presentation of the noise perturbation to further assess the extended, long-term persistence of vocal plasticity. In detail, Group 3 was recorded for 11 weeks (during which 108970 calls were collected) and Group 4 was recorded for 5 weeks (due to the much lower daily call rate of this group, 16157 calls). Figure 3 shows the change from baseline for each of the two groups, as computed for the mean of the centroid in bins of 5000 calls with an overlap of 500 calls between bins. As expected, and due to the fact that bat social calls are exclusively tied to complex social interactions, the difference from baseline fluctuated somewhat during this extended period of time, yet remarkably, never returned to baseline. Thus, the shift persisted for the entire duration of monitoring, i.e., on the time scale of months.

Analysis of the centroid during the last week of baseline and the week after noise presentation in terms of their variability offers insight into the magnitude of fluctuations. We thus assessed the variability for each group before and after the noise exposure, which revealed that although for two of the four groups the calls differed in their variance in the period after noise perturbation as compared to baseline, the differences for each group were small and not unique in one direction (more or less variable) (Supplementary Fig. 5 and Supplementary Tables 7 and 8). These findings strongly suggest that bats are capable of long-term and persistent vocal plasticity that is not altered in the absence of further changes in the acoustic environment or social constraints.

### Adult vocal plasticity persists across acoustic parameters

**Fig. 4:**
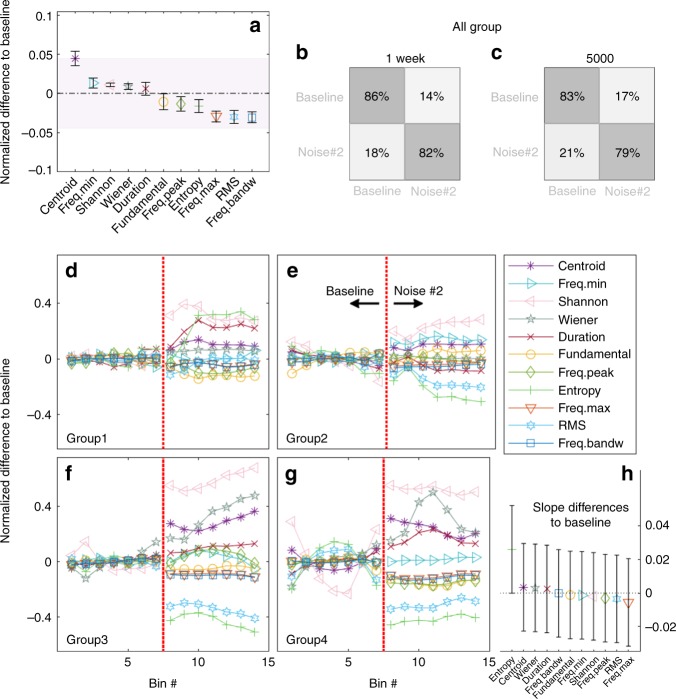
Stable effect of acoustic perturbation on multiple acoustic parameters. **a** mean shift compared to baseline across all groups for all extracted acoustic parameters (values are normalized to allow comparison); plots indicate the differences of the least square mean and their 95% confidence intervals. Though the strongest absolute shift is seen for the centroid, shifts are observed for other parameters as well; for example, frequency bandwidth (freq.bandw), RMS (root mean square) and maximum frequency (freq.max), indicating an overall effect of noise on acoustic call parameters. Peak frequency and minimum frequency are abbreviated with freq.peak and freq.min, respectively. The values and their significance for the various parameters are shown in Supplementary Table 2. **b**, **c** an unbiased classifier can correctly classify calls according to their baseline or noise condition. Results shown are for all groups for noise #2 based on the summarized 1-week data set (**b**) or the summarized 5000-data set for each condition (**c**). **d**–**g** development of the shifts for each parameter for each group is plotted in terms of difference to baseline. Each bin number is calculated over 25% of all calls recorded for a condition and group and a sliding window of 10% of all calls. The vertical red dotted line indicates time during baseline before noise exposure (left of the red dotted line) and after acoustic perturbation (right of the red dotted line). For each group, the shifts in acoustic parameters from baseline are visible but they remain stable over time, resulting in fairly flat lines. **h** this was verified by calculating the mean change in slopes on time between summarized baseline and noise #2 data set. The error bars are 99.55% confidence intervals of the mean difference in slopes, Bonferroni-corrected for 11 parameters. None of the parameters’ slopes after noise manipulation statistically differed from their baseline slope (randomized complete block ANOVA)

While our manipulation was designed to target changes in the spectral centroid of the bats’ vocalizations, it had no explicit constraints that would prevent the bats from also modifying other features of their calls. To assess this possibility and importantly, to examine the persistence of such potential changes, we computed 10 additional features, which have been used previously to characterize Egyptian fruit bat social calls^31^. Figure 4a shows the sorted mean shift of these acoustic features for summarized calls measured after the cessation of the acoustic perturbation (comparison was done with summarized calls during the last week of baseline). Consistent with the results presented above, the centroid showed the largest normalized shift. Importantly however, we found that many of the other acoustic features of the bats’ vocalizations also appeared to have changed (Fig. 4a, see Methods, Supplementary Fig. 6 and Supplementary Tables 1–4). To examine whether such a global change might allow us to distinguish the vocalizations of the bats before and after the manipulation, we used an unbiased classifier (Matlab 2017b ClassificationLearner Toolbox, ensemble classifier Bagged Trees) on the summarized data sets. Indeed, we found that when considering all 11 acoustic parameters (Supplementary Table 4), 86% and 82% of all calls recorded for all groups in the 1-week data sets (419 baseline bins and 483 bins after manipulation), could be correctly assigned as belonging to the 1-week period before and after the noise manipulation, respectively (Fig. 4b). Similarly, when applying our analysis to the 568 bins (4 groups × 71 bins × 2 conditions) recorded before and after the same manipulation, we found that the unbiased classifier could correctly assign the calls as belonging to the calls recorded before and calls after the manipulation with accuracies of 83% and 79%, respectively (Fig. 4c).

These results suggest that the groups of bats significantly changed the global acoustic structures of their calls but do not address whether these global changes were also persistent. Figure 4d–g, shows the evolution of each of the 11 acoustic parameters for each group separately during the baseline period (left half of each panel to the dotted red line) and following the cessation of the acoustic perturbation (right half of each panel to dotted red line). For all groups, the divergence of the acoustic parameters after the acoustic perturbation compared to baseline is visible, and importantly, stable. Looking across different groups, we estimated the change over time (a slope) for each parameter both before and after the manipulation through linear regression on the summarized data sets (see Methods). We found that the slopes remained around zero within each time-period, indicating stability of the calls both before as well as after the noise manipulation (Fig. 4h). The slopes were also not significantly different when comparing the baseline to the post-perturbation period (11 unadjusted *p*-values between 0.005 and 0.979, *n* = 4 groups × 11 parameters per group, randomized complete block ANOVA), suggesting that the bats’ call structures were equally stable before and after the manipulation.

Lastly, and to again account for the natural variation on the groups’ social calls, we extracted normalized mean values on a day-by-day basis for each group and for each of the 11 acoustic parameters and compared (across all groups) the daily values between baseline and the post-noise exposure period (Supplementary Fig. 7). In agreement with the results obtained for the centroid measure (Fig. 2e), we found that for many acoustic parameters the daily post-perturbation period values were significantly different from nearly all baseline days. Together, our results suggest that the changes following the noise perturbation affected the global acoustic structure of bat social calls and importantly, that these global changes were stable and persistent.

### Analysis of context-specific vocalizations

**Fig. 5:**
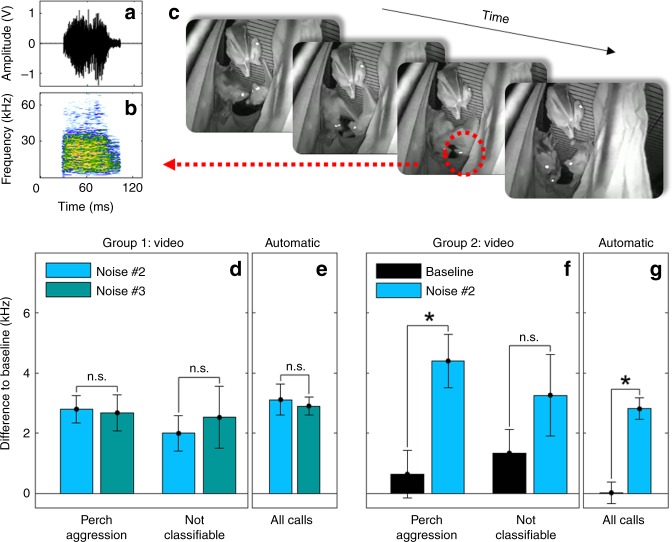
Analysis of context-specific vocalizations. **a**, **b** example of a bat vocalization is shown in the time (**a**) and frequency (**b**) domains. **c** image sequence as captured by the video cameras showing how a call is associated with a calling bat (red dotted circle) in a video frame and consequently categorized in terms of behavior type. **d**–**g** comparison of ‘Perch Aggression’ and ‘Not classifiable’ annotated calls to the automatic analysis shows that the observable shifts persist for specific behavioral contexts. Plotted is the mean centroid and the corrected standard error in terms of difference to summarized baseline data sets for summarized noise #2 and summarized noise #3 data sets for Group 1 (**d**, **e**) and for the summarized baseline and summarized noise #2 data sets for Group 2 (**f**, **g**). Notations above each bar indicate significance (*) or not (n.s.) when compared to the other condition, with *α* = 0.0045 and z-test through AR(1) model or SMA method

Egyptian fruit bat social calls are used exclusively for social interactions and their acoustic content is closely tied to the behavioral context in which they are emitted^17^. Thus, a potential confound in our results is that the bats did not actually modify the acoustic features of their vocalizations but instead avoided social behaviors that were associated with perturbed vocalizations. To assess this possibility, 15886 calls were recorded in conjunction with video monitoring (a call example and the associated video are depicted in Fig. 5a–c). These calls were manually annotated with respect to the observed behavior associated with each call (Fig. 5c). Annotation was made for calls recorded during the 2 weeks of baseline or for calls recorded during the week of silence after a noise cessation. This type of manual classification was conducted by unbiased observers who were blind to the details and goals of the experiment. For Group 1, 7868 calls produced after noise #2 and noise #3 were behaviorally annotated and for Group 2, 8018 calls produced during baseline and after noise #2 were behaviorally annotated. Based on previous studies^17^, the calls were categorized as belonging to six broad behavioral contexts (‘Perch Aggression’, ‘Sleep Aggression’, ‘Food Aggression’, ‘Mating Aggression’, ‘Positive Interaction’, ‘Positive Mating Interaction’). If a behavior could not be identified, it was categorized as ‘Not Classifiable’. Examples of different call types are shown in Supplementary Fig. 2 and their distribution is shown in Supplementary Fig. 8. Out of all manually annotated calls, 17% and 64% (for Group 1 and Group 2, respectively) could not be categorized due to the natural behavior of the bat groups (e.g., bats hanging very close together and occluding each other from view). Nevertheless, 77% and 34% of the calls were successfully categorized according to their associated behavior. Amongst all categorized calls, ‘Perch Aggression’ was by far the most prevalent in both groups, comprising a total of 82% and 94% of the calls produced by Groups 1 and 2, respectively. We thus focused our analysis on this call type (Supplementary Fig. 2). Calls categorized as ‘Perch Aggression’ and ‘Not Classifiable’ were sorted, summarized and the mean centroid was compared (Fig. 5d, f). For Group 1, the mean centroid of ‘Perch Aggression’ and ‘Not Classifiable’ calls from the period after noise #2 showed the same pattern as previously observed and were shifted relative to baseline by 2.798 ± 0.461 and 1.994 ± 0.591 kHz, respectively. A similar shift was observed for the period after noise #3 where the behaviorally annotated calls were shifted upward by 2.678 ± 0.593 and 2.525 ± 1.03 kHz. When comparing ‘Perch Aggression’ and ‘Not Classifiable’ calls between the two noise conditions, results were not significant relative to each other (*z* = 0.16 and *p* = 0.873, *n* = 136 bins for ‘Perch Aggression’, z-test through AR(1) model; *z* = 0.298, *R* = 0.08 and *p* = 0.674, *n* = 37 bins and 20000 iterations for ‘Not Classifiable’, SMA method, see Methods). When comparing the shift to baseline for the automatic call analysis, we found that the shift for these two noise conditions was 3.118 ± 0.545 and 2.883 ± 0.581 kHz (Fig. 5e) and these were also not significant relative to each other (*z* = 0.351 and *p* = 0.725, *n* = 325 bins, z-test through AR(1) model; Supplementary Fig. 1B). For Group 2, the mean centroids of ‘Perch Aggression’ and ‘Not Classifiable’ calls recorded during the baseline period were elevated by 0.636 ± 0.794 and 1.338 ± 0.781 kHz relative to the noise condition. This slight elevation was caused by calculating the mean over these selected calls which results in a slightly higher centroid in comparison to the mean centroid over all baseline calls from the automatic call analysis. More importantly, these specific types of calls were shifted even higher following the acoustic perturbation by 4.402 ± 0.878 and 3.263 ± 1.35 kHz relative to baseline (Fig. 5f) and the observed shift was significant for the ‘Perch Aggression’ calls (*z* = 3.388 and *p* = 0.002, *n* = 69 bins for ‘Perch Aggression, *z* = 1.214 and *p* = 0.225, *n* = 138 bins for ‘Not Classifiable’, z-test through AR(1) model). Though the ‘Not Classifiable’ baseline calls are not significantly different to the ‘Not Classifiable’ noise #2 calls, the difference in means follows the same trend as seen for the ‘Perch Aggression’ calls. Furthermore, this effect was comparable to the significant upward shift observed for this group when considering all calls (Fig. 5g, 2.795 ± 0.515 kHz). Combined, analysis of the manually classified calls based on social context reveals a similar pattern of spectral centroid shift, indicating that the observed shifts cannot be accounted for by changes in the social behavior of the group and associated call types. Rather, it is due to a global and stable adaptation of the produced vocalizations.

## Discussion

In the current study, we investigated the existence of persistent vocal plasticity in adult Egyptian fruit bats (*Rousettus aegyptiacus*) in response to noise exposure. Without exception, we found that all groups of bats, independent of group composition, environmental conditions and gender, adapted their vocalizations to minimize interference with the acoustic perturbation. Importantly, the observed shift persisted long after noise cessation (in some cases lasting for several weeks or months), which suggests a high degree of vocal plasticity that extends well into adulthood in this species. Our results complement previous studies of vocal plasticity in Egyptian fruit bats that have thus far been restricted to juveniles during development. The demonstration of long-term and persistent vocal plasticity in the adult bat presented in this study further helps establish the Egyptian fruit bat as a feasible and tractable mammalian model organism for studying the neural computations that support vocal plasticity in the adult mammalian brain.

Why do bats retain these unique vocal plasticity abilities as adults? Although this question remains unanswered, it is clear that bats are highly specialized in acoustic signaling. Bats are capable of remarkable vocal control, which up to now has been mostly studied in the domain of their “sixth-sense”, i.e., echolocation—a sensory modality used for orientation, navigation and prey acquisition^35,36^. Although bat species can differ greatly in their lifestyle, foraging strategy and prey items^37,38^, most of them rely on this extraordinary sensory modality which is highly adaptive in response to changes in their environment. For example, many bats echolocate more loudly, with longer calls and more often or may rapidly shift frequency components of their echolocation calls during increased ambient noise or to avoid jamming by conspecifics^32,39–44^. These changes, however, are typically short-term, i.e., restricted to the period of noise interference. In contrast, we have found that adult bats adapt the structure of their social calls in response to an external sound source by shifting spectral parameters of their vocalizations and importantly, maintain this shift after noise cessation. The persistence of the shift suggests that vocal plasticity exists beyond short-term changes or adolescence in bats and demonstrates that auditory inputs can drive these changes even in adulthood. The latter notion is consistent with emerging evidence indicating that bat social calls are learned^8,9,18,19^ and might therefore be associated with a high-degree of vocal plasticity that is retained throughout the bat’s life. This notion is ethologically conceivable, as animals who are acoustic specialists might benefit from a more persistent form of vocal adaptation, especially when exposed to an acoustic perturbation affecting the long-term effectiveness of their acoustic communication system.

A major question that remains is what mechanisms are used by bats to modify their vocalizations? According to the source-filter theory of sound production, vocalizations are the products of the vocal tract (tongue, lips, soft palate), which filter source signals originating in the syrinx (in birds) or larynx (in mammals)^45–47^. As the sound source determines the fundamental frequency through the vibration of the vocal folds, changes in the fundamental are possible via adjusting the sound source while changes in other spectral features occur through adapting the filter of the vocal tract. Comparing all extracted acoustic parameters between baseline and the noise condition in our study revealed that the centroid showed the largest shift, but changes were evident for almost all acoustic parameters. This, in turn, indicates a multifactorial, long-lasting effect induced by the presented acoustic perturbation on the call structure. An animal’s acoustic environment can deviate strongly in its signal-to-noise ratio in a dynamic fashion, enforcing evolved mechanisms that allow the system to deal with these types of variations under noisy conditions^13,32,43,44,48–51^. Thus, adult bats might retain a high level of control over both the source and filter and can select which aspects of their modification need to be adjusted based on the specific environmental conditions—a notion that is compatible with the stability observed across all extracted parameters.

In addition, it has been postulated that changes in the spectral features of vocalizations could be a by-product of the Lombard effect^27^—an increase in signal amplitude influenced by noise—where the increase in call amplitude results in the widening of the glottal width and a shortening of the vocal folds shifting spectral features upward^45^. This, however, is not the cause for the increased centroid in our study since we see no increase in amplitude after noise cessation. On the contrary, the amplitude (calculated from the root mean square) was reduced in comparison to baseline by 1 dB (Supplementary Table 4) and was negatively correlated with the centroid shift, but this correlation is not significant (Supplementary Fig. 9A). The additional measurement of the peak-to-peak amplitude (Supplementary Table 9) showed a similar downward shift after noise perturbation—consistent with the trend found for the RMS—indicating that the call amplitude did not increase in its peak or average amplitude. A prior study investigating the Lombard effect on echolocation calls of bats found that frequency shifts in response to noise perturbation could occur independently of changes in amplitude^44^. Nevertheless, the classical definition of the Lombard effect refers to changes in vocalization structure not only in response to an increase in ambient noise levels, but more importantly, during the noise exposure itself^48,52^. The observed shifts in centroid found in the current study were always measured in complete silence and for an extended period of time after noise cessation. Studies in songbirds^53^ and cetaceans^54,55^ showing long-term changes in vocalizations in response to noise (over periods of years to decades) have also been linked to the Lombard effect. But, since these studies were conducted in the free field, they did not control for the persistence or disruption of the noise conditions. Consequently, the shifts measured in the current study in silence and for an extended period of time (multiple weeks) after noise cessation cannot be attributed to the Lombard effect, at least not according to its classical definition in the literature. However, it is important to consider that since the Lombard effect is one of multiple mechanisms underlying vocal plasticity, the underlying mechanisms may not be mutually exclusive.

It has also been shown that habitat or social context can shape vocal signals^56–58^ (e.g. developing *R. aegyptiacus’* social calls can be influenced by calls of an entire social group, known as ‘crowd vocal learning’). Such vocalizations could be termed vocal ‘dialects’^19,59^ or act as vocal group signatures allowing for group membership and possibly origin to be discerned^18,59–63^. The bats in the present study all experienced the same noise perturbation and were not exposed to calls by other conspecifics during the recording time, potentially resulting in a group call signature. Thus, when three of the groups had been recaptured after release into a large colony (comprising hundreds of bats), the differences in the measured centroid (consistent with the notion that social calls are highly influenced by acoustic environment) were revealed (Supplementary Fig. 10). It is important to note that the different groups were released to colonies of dramatically different structures in terms of both numbers and sex (e.g. an all-male vs. mixed-gender colony) and future studies in which the colony social structure is characterized and controlled will be important for studying the extent of vocal plasticity in a naturalistic, large-scale social group context.

Together, our results demonstrate persistent vocal plasticity in adult Egyptian fruit bats, thus supporting their potentially important role as a model system for vocal production and learning. Alongside recent technological developments for interrogating the neural circuits of these remarkable mammals, these findings could enable the long-awaited study of the neural circuits supporting vocal plasticity in the mammalian brain.

## Methods

### Animals

*Rousettus aegyptiacus* is a megachiropteran, Old-World fruit bat found throughout Africa and the Middle-East. Twenty adult bats were removed from breeding colonies at the University of California, Berkeley, and separated into four groups of six or four bats per group. Group 1 consisted of three males and three females (which gave birth to one pup each toward the end of the experiment), Group 2 of six males, and Groups 3 and 4 of four males each. Each group was housed continuously in a cage with a daily free fly time of 1 h. Food (a fruit-vegetable mix consisting of apples, pears, melons, grapes, plums, kale, carrots and sweet potatoes) and water were given ad libitum. All experimental procedures complied with all relevant ethical regulations for animal testing and research and were approved by the Institutional Animal Care and Use Committee of the University of California, Berkeley.

### Group setup

Based on published data by Prat et al.^8,17,31^ and personal observations we know that these bats do not call in isolation but in groups of bats (>3 animals) where most vocal interactions occur pairwise. Group 1 was chosen to consist of six animals and balanced in gender. During the course of the experiment, all three females became pregnant and gave birth. As the pups’ isolation calls are fairly high in frequency content^8,17^ the observed upward shift in centroid could have been induced by such calls. Group 2 was therefore chosen to consist of only males to avoid this potential confound. To test different environmental conditions, Groups 3 and 4 were set in an acoustically quieter and shielded environment (sound boxes, see below). The smaller size of the cages and enclosures used in these experiments limited the number of animals in each group to four (compared to six in the first two groups). Initially, Groups 3 and 4 had consisted of three females each, but as both female groups barely communicated vocally (<80 call triggers per day) already during the baseline period, these groups were discontinued before any noise manipulation and instead rearranged to consist of only males (four individuals per group). Importantly, none of the bats had experienced any type of playback prior to this study.

### Experimental setup

Groups 1 and 2 were housed in a metal cage (81 × 48 × 94 cm) with a wire spacing of 1.3 cm set at an overall height of 163 cm (Fig. 1a) and positioned in a corner of a larger housing room. The cage was surrounded by felt curtains to dampen echoes. Two loudspeakers (Discovery Tweeter R2604/833000, Scanspeak, Videbæk, Denmark) were positioned at a height of 120 cm and 10 cm distance with respect to the front and the back of the cage. At the front of the cage, one microphone (custom built from Knowles SPU0410LR5H-QB, Itasca, IL, USA) was placed at a distance of 10 cm and height of 120 cm. An additional USB camera (Megapixel IP camera, ELP, Ailipu Technology Co. Ltd, Shenzhen, Guangdong, China) was mounted to a side of the cage later in the course of the experiment and used to record (motion-based, triggered video recordings) the events in the cage. Groups 3 and 4 were housed in a plastic mesh cage (26 × 51 × 30 cm) set in an acoustic chamber (44 × 76 × 60 cm) lined with acoustic foam, (Fig. 1b). The microphone was positioned in a back corner 10 cm from the ceiling, pointing towards the cage, and the speaker was mounted in the middle of the ceiling facing downwards towards the cage. The USB camera was affixed in the middle of the cage door with the cage being in its field of view. Microphones were connected directly and the speakers via a stereo amplifier (Servo 120 A, Samson Technologies, Hicksville, NY, USA) to an A/D-D/A converter (UltraLite-mk3, MOTU, Cambridge MA, USA) with a sampling rate of 192 kHz, which sent and received its signals from a PC. Recordings were triggered through a combination of signal duration and loudness. Recording and playback was controlled via the Soundmexpro (HörTech gGmbH, Oldenburg, Germany) toolbox and Matlab (Matlab R2011a, MathWorks, Natick, MA).

### Procedure

Each group of bats was monitored for 1 week while they became accustomed to the new environment and social arrangement. After this habituation, their vocal activity was recorded for 2 weeks to establish a baseline call behavior. Following this time period, playback of continuous background noise (the acoustic perturbation) commenced. The bats were exposed for 2 weeks to a background noise followed by 1 week of silence. This was repeated for up to two or three different noise conditions for Groups 2 or 1, respectively.

### Stimuli

Different noise conditions were defined through their spectral tilts (Fig. 1c and Supplementary Fig. 1A). This was accomplished by designing an impulse response with a defined level decrease per octave (−3, −6, and −12 dB per octave); a steeper downward slope results in less activation of higher frequency auditory filters. This resulted in three different noise conditions: first noise condition (noise #1), with a shallower slope and the most energy between 8–27 kHz, the second noise condition (noise #2), with a steeper slope and its energy shifted towards lower frequencies (4–20 kHz) and the third noise condition (noise #3), with the steepest slope and energy mainly in the low frequency range (below 5 kHz). By convolving white noise with the impulse response, the spectral tilt is imposed onto the presented noise. The speaker’s frequency response was not corrected as it matched the noise’s spectral shape. The background noise was presented at 45–50 dB SPL (as used by Luo and Wiegrebe^32^) and was continuously active for the two playback weeks. Groups 1 and 2 first experienced noise #1 followed by noise #2. Group 1 was furthermore exposed to noise #3. Groups 3 and 4 were only exposed to noise #2. For Groups 1 and 2, noise was silenced for 1 week between the two different noise presentations (noise #1 followed by noise #2) to observe long-term effects on the bats’ vocalizations. After the last noise condition and the subsequent week of silence, Groups 1 and 2 were released back to their original colony. Before returning them to the colony, Groups 3 and 4 were recorded for an additional 11 or 5 weeks, respectively, to measure whether the bats would adapt their calls back to their original baseline or retain their adapted calls. Group 4 was released after 5 weeks due to a low call rate and unchanged call parameters. Group 3 was released after 11 weeks with no apparent reversal in call parameters to their baseline. Groups 2, 3, and 4 were recaptured after being back in the colony for 1 month, brought back to their previous experimental setups and recorded for 1 month. The purpose was to investigate whether the bats would adapt their calls back to their original baseline after being exposed to a more natural acoustic environment.

All noise conditions were played via the setup speaker(s) and recorded with a calibration microphone (¼″ prepolarized free-field microphone 40BE and ¼″ CCP preamplifier 26CB, G.R.A.S. Sound & Vibration A/S, Holte, Denmark) with a flat frequency response up to 100 kHz to ensure correct frequency presentation of the noise. Amplitude levels were adapted with the help of a sound calibrator (1 kHz at 94 dB, Tenma 72–7260, Leeds, UK).

### Call detection and analysis

A Matlab-based, custom designed code automatically cut social calls from other cage-related noise in the recordings. Recordings were low-pass filtered and peaks in the sound envelope above a certain threshold were detected, resulting in an initial signal cut. Duration and root mean square thresholds determined whether a detected signal was a call or not. An additional custom designed function evaluated the envelope of the detected signal which was checked against a duration threshold for a second time. Consequently, detected calls were filtered (hamming filter) and then analyzed according to their duration, root mean square (RMS), entropy, peak frequency, minimum and maximum frequencies, frequency band width, centroid, fundamental, Wiener and Shannon entropy. The peak frequency was detected in the call’s magnitude spectrum, the minimum and maximum frequencies were at the ±25 dB cutoff points of the peak frequency, and the frequency bandwidth was the difference between these two cutoff points. The centroid, fundamental and Wiener and Shannon entropy were calculated on the median over call snippets (10% of entire call length) and a sliding window of 9% of the entire call length over the entire call. The centroid was measured by dividing the sum of a set frequency range (for Groups 1 and 2, 0.5–60 kHz and Groups 3 and 4, 0.5–40 kHz) by the sum of the power spectrum of the frequency range. For extraction of the fundamental the Matlab-based functions ‘spCorr’ and ‘spPitchCorr’ were adapted (with frequency limits set to 0.5 and 10 kHz). For evaluation of the Wiener entropy, the Matlab-based function ‘spectral_flatness’ was used and for the Shannon entropy and the entropy (calculated over the spectrum and the time signal) the Matlab-based function ‘wentropy’ was employed. The RMS was the root mean square of the call amplitude in voltage. For further analysis, only calls with a centroid below 80 kHz and a fundamental between 0.5 and 5 kHz were considered valid, as this reduced the risk of including cage noise.

### Data summarization

As the Lilliefors test revealed that the data sets were not Gaussian distributed at a 5% significance level, we applied a summarization method on data sets. This was done by averaging every 70 calls, resulting in a number of observation bins (e.g. 6348 baseline calls for Group 1 resulted in 90 observation bins, each consisting of a mean over 70 calls). The resulting errors of these means are closer to a normal distribution than when calculated over the non-summarized data. The Lilliefors test on the summarized data verified its normal distribution. This summarization into observation bins was applied to each condition or time-period (baseline and after noise manipulation) and group. Due to overall smaller sample sizes of the half data-sets (of the 1-week and 5000 calls) and the manually classified calls we averaged over 50 calls and 35 calls, respectively, to ensure at least about 30 observation bins for each data set (except for one exception see below). Statistics (see below) was applied on summarized data sets.

For normalization, each group data was normalized by first subtracting from the mean of the baseline observation bins and then dividing by the maximum of the absolute value for the baseline observation bins.

### Statistics

When comparing baseline and post-perturbation conditions within one group, we applied a *z*-test for comparing between two time-series.; this test is described in section 15.2 of Ramsey and Schafer^64^. The test assumes each time series has a constant mean, that observations within the time series exhibit 1st order autocorrelation (AR(1)), and that the autocorrelation and variance is the same for both time series. We refer to this test as the “z-test through AR(1) model”. For each group we report the estimated AR(1) correlation, *r*_*1*_, and pooled standard deviation in Supplementary Tables 5 and 6, and the differences of the least square means and corrected standard errors (mean ± corrected standard error) in the main results section.

The smaller sample size of the video annotated data set for Group 1 ‘Not Classifiable’ calls resulted in less than 30 observation bins per noise #2 and noise #3 condition. To statistically analyze these short, autocorrelated data sets we applied the Simulation Modelling Analysis (SMA) developed by Borckardt and Nash^65^ for small sets of single-subject data collected over time. In brief, SMA first estimates the means of each condition and a common AR(1) correlation, *r*_*1*_. Then, under a null-model of no difference in means, simulates many datasets under the original data structure, with observations having *r*_1_ correlation. The resulting empirical *p*-value is the proportion of simulated datasets having differences in means that were at least as extreme as the originally observed difference in means.

For comparing means between baseline and post-perturbation conditions from multiple groups, we applied a mixed linear model, which assumed observations within each group-condition combination had a common variance and AR(1) correlation. We estimated the denominator degrees of freedom for the resulting *t*-tests according to the Kenward-Roger^66^ method. For each time-period and group we report the differences of the least square means and the standard errors in the main result section.

For each parameter a linear regression extracted the slope of the measure over time for baseline and noise #2. The differences between baseline and noise #2 slope for each parameter was fitted by a randomized complete block ANOVA test where group was a random and parameter a fixed factor. This analysis accounts for the natural relationship between baseline and noise #2 slopes and for the correlation of parameters from the same group.

All values were obtained by combining results from the SAS statistical software (SAS University edition, SAS Institute Inc., Cary, U.S.A.) or from the SMA free software (SMA–Version 11.10.16, Copyright J. J. Borckhardt) with customized Matlab codes. Additionally, the alpha level of 5% is Bonferroni corrected to account for multiple comparisons (11 call parameters → 0.05/11 = 0.0045).

### Classifier

The Bagged Trees classifier in the Matlab classificationLearner toolbox uses the Breiman’s ‘random forest’ algorithm (for reference, see Breiman et al.^67^). We note however, the classifier assumes independence among all observations and the observed 1st order autocorrelation for each parameter data set is between 0.206 and 0.523.

### Behavioral analysis

Group 1 calls recorded during the week of silence after exposure to noise #2 and #3 and Group 2 calls recorded during the baseline period and during the week of silence after noise #2, were selected for annotation based on behavior recorded in the videos by student volunteers. These unbiased observers were blind to the specifics of the project design and goal. A valid annotation meant that a call could be reliably synchronized with a video sequence and the behavioral content identified. Due to the camera’s field of view, a select few identifiable behaviors were chosen for annotation. These behavior types were based on a previous behavioral classification study done for the same species (for details see Prat et al.^17^).

Perch aggression: aversive behavior of one bat to another when in close vicinity to each other

Sleep aggression: aversive behavior when at least one bat had been sleeping

Food aggression: squabble between bats with food involved

Mating aggression: aversive behavior between a male and female during mating (attempts)

Positive interaction: non-aversive behavior between bats, typically during grooming

Positive mating interaction: non-aversive behavior between bats during mating (attempts)

The goal of this manual analysis was to verify that any change in the bats’ vocalization parameters was not due to a change of call type induced by a change in behavior. If the call type could not be determined it was termed ‘Not Classifiable’. Due to the nature of the setup and recording procedure, a large amount of the audio recordings was cage-related noise. The manual analysis of the calls therefore additionally provided proof that only a small percentage of cage-related noise contaminated the automatic post call analysis.

### Reporting summary

Further information on research design is available in the Nature Research Reporting Summary linked to this article.

## Supplementary information

Supplementary Information

Reporting Summary

## Data Availability

All data supporting the findings in this study is available from the corresponding author upon request.

## Code availability

All code designed for analyzing the data in this study is available from the corresponding author upon request.

## References

[1] Guenther FH (1994). A neural network model of speech acquisition and motor equivalent speech production. Biol. Cybern..

[2] Callan DE, Kent RD, Guenther FH, Vorperian HK (2000). An auditory-feedback-based neural network model of speech production that is robust to developmental changes in the size and shape of the articulatory system. J. Speech, Lang. Hear. Res..

[3] Oller DK, Eilers RE (1988). The role of audition in infant babbling. Child Dev..

[4] Reichmuth C, Casey C (2014). Vocal learning in seals, sea lions, and walruses. Curr. Opin. Neurobiol..

[5] Petkov CI, Jarvis ED (2012). Birds, primates, and spoken language origins: behavioral phenotypes and neurobiological substrates. Front. Evol. Neurosci..

[6] Doupe AJ, Kuhl PK (1999). Birdsong and human speech: common themes and mechanisms. Annu. Rev. Neurosci..

[7] Stoeger AS, Manger P (2014). Vocal learning in elephants: neural bases and adaptive context. Curr. Opin. Neurobiol..

[8] Prat Y, Taub M, Yovel Y (2015). Vocal learning in a social mammal: Demonstrated by isolation and playback experiments in bats. Sci. Adv..

[9] Esser KH (1994). Audio-vocal learning in a non-human mammal: the lesser spear-nosed bat Phyllostomus discolor. Neuroreport.

[10] Janik VM (2014). Cetacean vocal learning and communication. Curr. Opin. Neurobiol..

[11] Takahashi D. Y., Fenley A. R., Teramoto Y., Narayanan D. Z., Borjon J. I., Holmes P., Ghazanfar A. A. (2015). The developmental dynamics of marmoset monkey vocal production. Science.

[12] Brainard MS, Doupe AJ (2013). Translating birdsong: songbirds as a model for basic and applied medical research. Annu. Rev. Neurosci..

[13] Tumer EC, Brainard MS (2007). Performance variability enables adaptive plasticity of ‘crystallized’ adult birdsong. Nature.

[14] Reiner A, Yamamoto K, Karten HJ (2005). Organization and evolution of the avian forebrain. Anat. Rec. - Part A Discov. Mol., Cell., Evolut. Biol..

[15] Vernes SC (2017). What bats have to say about speech and language. Psychon. Bull. Rev..

[16] Knoernschild M (2014). Vocal production learning in bats. Curr. Opin. Neurobiol..

[17] Prat Y, Taub M, Yovel Y (2016). Everyday bat vocalizations contain information about emitter, addressee, context, and behavior. Sci. Rep..

[18] Boughman JW (1998). Vocal learning by greater spear-nosed bats. Proc. R. Soc. B-Biol. Sci..

[19] Prat, Y., Azoulay, L., Dor, R. & Yovel, Y. Crowd vocal learning induces vocal dialects in bats: playback of conspecifics shapes fundamental frequency usage by pups. *PLoS Biol*. **15**, e2002556 (2017). 10.1371/journal.pbio.2002556 PMC5663327 29088225

[20] Austad, S. N. & Fischer, K. E. Mammalian aging, metabolism, and ecology: evidence from the bats and marsupials. *J. Gerontol*. **46**, B47–53 (1991). 10.1093/geronj/46.2.b47 1997563

[21] Kwiecinski, G. G. & Griffiths, T. A. Rousettus egyptiacus. *Mamm. Species*. **611**, 1–9 (1999).

[22] Zhang Wujie, Yartsev Michael M. (2019). Correlated Neural Activity across the Brains of Socially Interacting Bats. Cell.

[23] Yartsev MM, Witter MP, Ulanovsky N (2011). Grid cells without theta oscillations in the entorhinal cortex of bats. Nature.

[24] Yartsev MM, Ulanovsky N (2013). Representation of three-dimensional space in the Hippocampus of flying bats. Science.

[25] Bradbury, J. W., Vehrencamp, S. L., Bradbury, J. W. & Vehrencamp, S. L. *Principles of animal communication*. *Principles of animal communication*. (Sinauer Associates, Sunderland, MA 1998).

[26] Wiley R (2006). Animal communication: signal detection. Encycl. Lang. Linguist. Second Ed. Vol. 1.

[27] Hotchkin C, Parks S (2013). The Lombard effect and other noise-induced vocal modifications: insight from mammalian communication systems. Biol. Rev..

[28] Brumm, H. & Slabbekoorn, H. Acoustic communication in noise. in *Advances in the Study of Behavior*, Vol 35 (eds Slater, P. J. B., Snowdon, C. T., Brockmann, H. J., Roper, T. J. & Naguib, M.) 151–209 (Academic Press, Cambridge, MA 2005).

[29] Slabbekoorn H (2013). Songs of the city: noise-dependent spectral plasticity in the acoustic phenotype of urban birds. Anim. Behav..

[30] Koay G, Heffner RS, Heffner HE (1998). Hearing in a megachiropteran fruit bat (Rousettus aegyptiacus). J. Comp. Psychol..

[31] Prat, Y., Taub, M., Pratt, E. & Yovel, Y. Data descriptor: an annotated dataset of egyptian fruit bat vocalizations across varying contexts and during vocal ontogeny. *Sci. Data* **4**, 170143 (2017). 10.1038/sdata.2017.143 PMC5625625 28972574

[32] Luo J, Wiegrebe L (2016). Biomechanical control of vocal plasticity in an echolocating bat. J. Exp. Biol..

[33] Luo J, Lingner A, Firzlaff U, Wiegrebe L (2017). The Lombard effect emerges early in young bats: implications for the development of audio-vocal integration. J. Exp. Biol..

[34] Schubert, E., Wolfe, J. & Tarnopolsky, A. Spectral centroid and timbre in complex, multiple instrumental textures. in *Proc. 8th International Conference on Music Perception and Cognition*. 654–657 (Causal productions, Evanston, IL 2004).

[35] Moss CF, Surlykke A (2001). Auditory scene analysis by echolocation in bats. J. Acoust. Soc. Am..

[36] Neuweiler, G. *The Biology of Bats*. (Oxford University Press, Oxford United Kingdom 2000).

[37] Denzinger, A. & Schnitzler, H. U. Bat guilds, a concept to classify the highly diverse foraging and echolocation behaviors of microchiropteran bats. *Front. Physiol*. **4**, 164 (2013). 10.3389/fphys.2013.00164 PMC3699716 23840190

[38] Genzel D, Yovel Y, Yartsev MM (2018). Neuroethology of bat navigation. Curr. Biol..

[39] Amichai, E., Blumrosen, G. & Yovel, Y. Calling louder and longer: how bats use biosonar under severe acoustic interference from other bats. *Proc. R. Soc. B Biol. Sci*. **282**, 20152064 (2015). 10.1098/rspb.2015.2064 PMC4707756 26702045

[40] Ulanovsky N, Fenton MB, Tsoar A, Korine C (2004). Dynamics of jamming avoidance in echolocating bats. Proc. R. Soc. B-Biol. Sci..

[41] Hage SR, Metzner W (2013). Potential effects of anthropogenic noise on echolocation behavior in horseshoe bats. Commun. Integr. Biol..

[42] Luo, J., Goerlitz, H. R., Brumm, H. & Wiegrebe, L. Linking the sender to the receiver: vocal adjustments by bats to maintain signal detection in noise. *Sci. Rep*. **5**, 18556 (2015). 10.1038/srep18556 PMC4686984 26692325

[43] Tressler J, Smotherman MS (2009). Context-dependent effects of noise on echolocation pulse characteristics in free-tailed bats. J. Comp. Physiol. a-Neuroethol. Sens. Neural Behav. Physiol..

[44] Hage SR, Jiang T, Berquist SW, Feng J, Metzner W (2013). Ambient noise induces independent shifts in call frequency and amplitude within the Lombard effect in echolocating bats. Proc. Natl Acad. Sci. USA.

[45] Titze IR (1989). On the relation between subglottal pressure and fundamental frequency in phonation. J. Acoust. Soc. Am..

[46] Lieberman P (2007). The Evolution of Human Speech: Its Anatomical and Neural Bases. Curr. Anthropol..

[47] Elemans CPH, Zaccarelli R, Herzel H (2008). Biomechanics and control of vocalization in a non-songbird. J. R. Soc. Interface.

[48] Brumm H, Zollinger A (2011). The evolution of the Lombard effect: 100 years of psychoacoustic research. Behaviour.

[49] Brumm H, Slabbekoorn H (2005). Acoustic Communication in Noise. Adv. Study Behav..

[50] Hotchkin, C. F., Parks, S. E. & Weiss, D. J. Noise-induced frequency modifications of tamarin vocalizations: implications for noise compensation in nonhuman primates. *PLoS ONE* **10**, e0130211 (2015). 10.1371/journal.pone.0130211 PMC4479599 26107515

[51] Lazerte, S. E., Slabbekoorn, H. & Otter, K. A. Learning to cope: vocal adjustment to urban noise is correlated with prior experience in black-capped chickadees. *Proc. R. Soc. B Biol. Sci*. **283**, 938–949 (2016). 10.1098/rspb.2016.1058 PMC4936046 27358372

[52] Luo Jinhong, Hage Steffen R., Moss Cynthia F. (2018). The Lombard Effect: From Acoustics to Neural Mechanisms. Trends in Neurosciences.

[53] Patricelli G, Blickley JJL (2006). Avian communication in urban noise: causes and consequences of vocal adjustment. Auk.

[54] Foote AD, Osborne RW, Hoelzel AR (2004). Whale-call response to masking boat noise. Nature.

[55] Parks SE, Clark CW, Tyack PL (2007). Short- and long-term changes in right whale calling behavior: the potential effects of noise on acoustic communication. J. Acoust. Soc. Am..

[56] Nicholls JA, Goldizen AW (2006). Habitat type and density influence vocal signal design in satin bowerbirds. J. Anim. Ecol..

[57] Schmidt AKD, Riede K, Romer H (2011). High background noise shapes selective auditory filters in a tropical cricket. J. Exp. Biol..

[58] Bohn KM, Smarsh GC, Smotherman M (2013). Social context evokes rapid changes in bat song syntax. Anim. Behav..

[59] Esser KH, Schubert J (1998). Vocal dialects in the lesser spear-nosed bat Phyllostomus discolor. Naturwissenschaften.

[60] Boughman JW (1997). Greater spear-nosed bats give group-distinctive calls. Behav. Ecol. Sociobiol..

[61] Elowson AM, Snowdon CT (1994). Pygmy marmosets, Cebuella pygmaea, modify vocal structure in response to changed social environment. Anim. Behav..

[62] Rukstalis M, Fite JE, French JA (2003). Social change affects vocal structure in a callitrichid primate (Callithrix kuhlii). Ethology.

[63] Watson SK (2015). Vocal learning in the functionally referential food grunts of chimpanzees. Curr. Biol..

[64] Ramsey, F. L. & Schafer, D. W. *The Statistical Sleuth: A Course in Methods of Data Analysis*. (Duxbury Press, Pacific Grove, CA 2002).

[65] Borckardt Jeffrey J., Nash Michael R. (2014). Simulation modelling analysis for small sets of single-subject data collected over time. Neuropsychological Rehabilitation.

[66] Kenward MG, Roger JH (2009). An improved approximation to the precision of fixed effects from restricted maximum likelihood. Comput. Stat. Data Anal..

[67] Breiman L (2001). Random forests. Mach. Learn.

