# Supplementary material for: Long-term and persistent vocal plasticity in adult bats
Source: Nat Commun. 2019 Jul 29;10:3372. doi: 10.1038/s41467-019-11350-2 (PMC6662767; doi:10.1038/s41467-019-11350-2)
Supplement: Supplementary file 1 — Supplementary Information [file 41467_2019_11350_MOESM1_ESM.pdf]

Supplementary Materials for

Long-term and Persistent Vocal Plasticity in Adult Bats

Daria Genzel, Janki Desai, Elana Paras and Michael M. Yartsev

- This supplementary material contains Supplementary Figs 1 – 10 and Tables 1-9

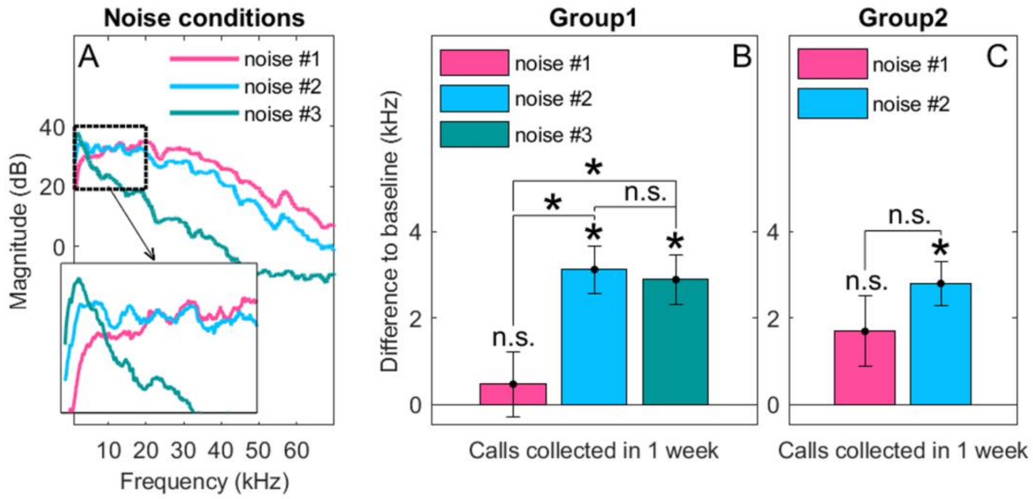

Supplementary Figure 1: Noise magnitude spectra and resulting shifts for Groups 1 and 2
(A) plotted are the magnitude spectra of the three noise conditions. The difference in the frequency distributions (more energy in higher, mid and low frequency regions) is visible, each targeting different frequency areas of either the bats' social calls or their most sensitive hearing range. The inset displays a close-up of the frequency area indicated by the dotted black lines. (B-C) the mean centroid and standard error for each summarized noise data set is plotted in terms of difference to baseline. (B) shows the shifts for Group 1 and (C) for Group 2. Plotted are mean centroid shifts and corrected standard errors after noise cessation as compared to baseline. Only Group 1 was exposed to a background noise with more energy in the lower frequency range (noise #3) and recorded for one week after noise cessation (17070 calls recorded). The mean centroid of the summarized calls of noise #3 shifted significantly (z=4.966 and p<0.001, n=334 bins, z-test through AR(1) model) by 2.883 ± 0.581 kHz compared to the bats' baseline and significantly (z=2.995 and p=0.003, n=293 bins, z-test through AR(1) model) by 2.407 ± 0.804 kHz compared to noise #1, but not significantly (z=0.351 and p=0.725, n=325 bins, z-test through AR(1) model) by -0.212 ± 0.605 kHz compared to noise #2. Notations above each bar indicate significance (*) or not (n.s.) when compared to baseline or another noise condition, with α=0.0045 and z-test through AR(1) model.

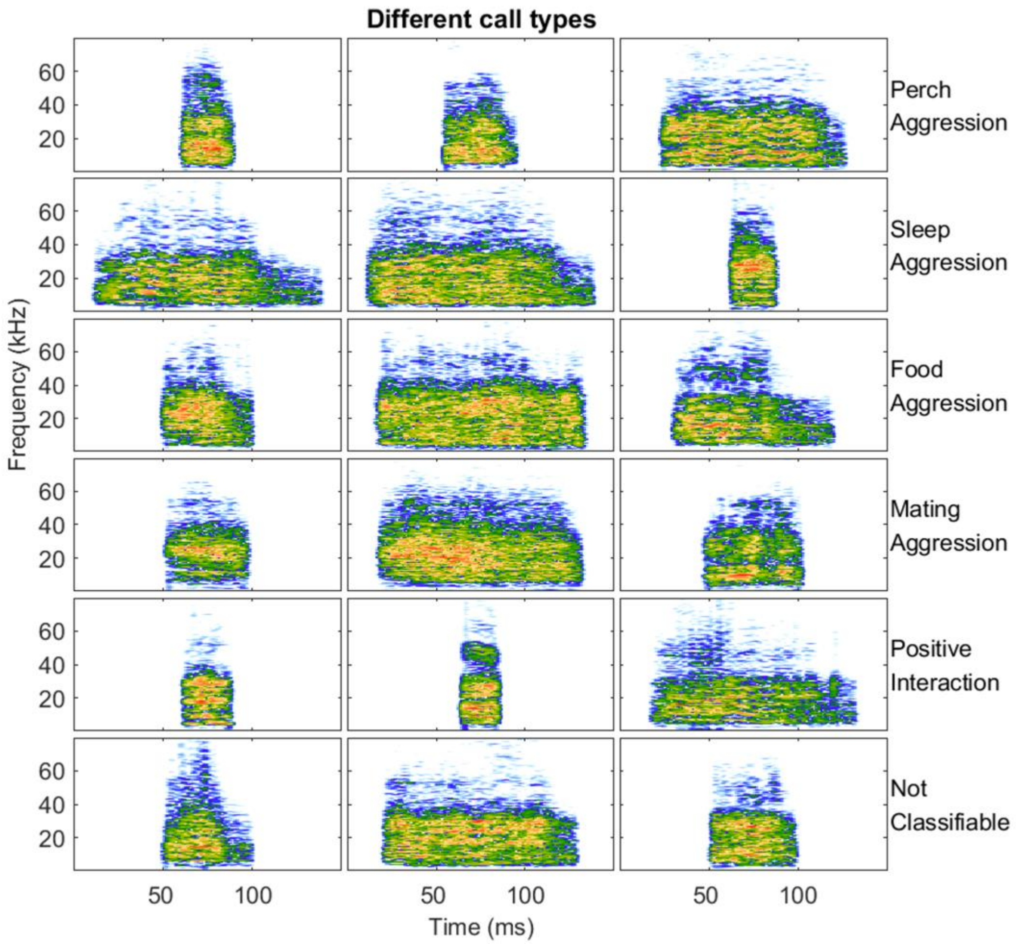

Supplementary Figure 2: Examples of different call types

Depicted are three examples each for different types of agonistic calls ('Perch aggression', 'Sleep Aggression', 'Food Aggression', 'Mating Aggression') and three examples for calls found in so-called 'Positive Interactions'. Three examples of calls which could not be annotated ('Not Classifiable') due to, for example, occlusion of the calling bat, are depicted in the bottom row and, in terms of their call structure, are very comparable to the identified call types. Colors code amplitude scale, with lower values in blue and higher values in red.

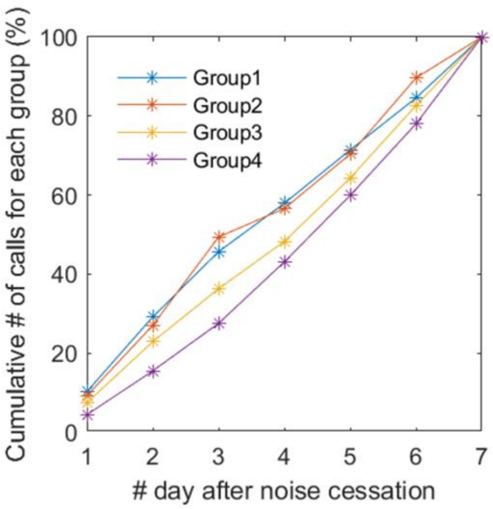

Supplementary Figure 3: Call rate stability

To check for stability of call rate over time, the cumulative number of calls during the week after noise cessation is plotted for each group separately on a day-by-day basis. As all slopes are fairly similar, the call rate for each group did not vary during the week after noise cessation.

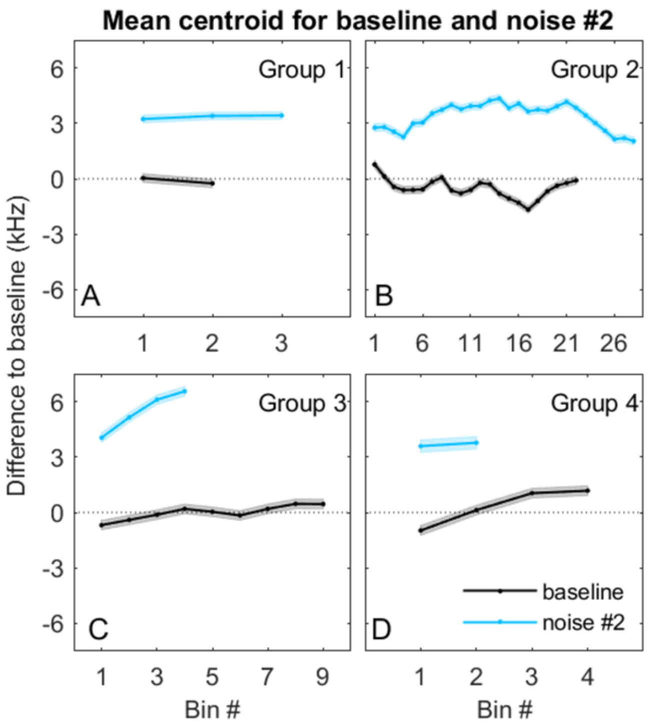

Supplementary Figure 4: Development of centroid shift

Depicted is the development of the mean centroid during the baseline and noise #2 1-week measurement periods (individual groups are shown in separate panels). Data is presented in terms of difference from the mean baseline. The standard error is indicated by the shaded area. A bin number is generated by calculating the mean over 5000 calls and a sliding window of 500 calls, except for the baseline of Group 1 and noise #2 for Group 4, where due to low call numbers a bin size of ~3000 calls was used. In general, lower call rates result in lower bin numbers. The mean centroid fluctuation for the baseline and after noise cessation are comparable, but more importantly, the fluctuations do not overlap. This demonstrates that changes in response to noise perturbation are above fluctuations of individual groups over the entire measurement period.

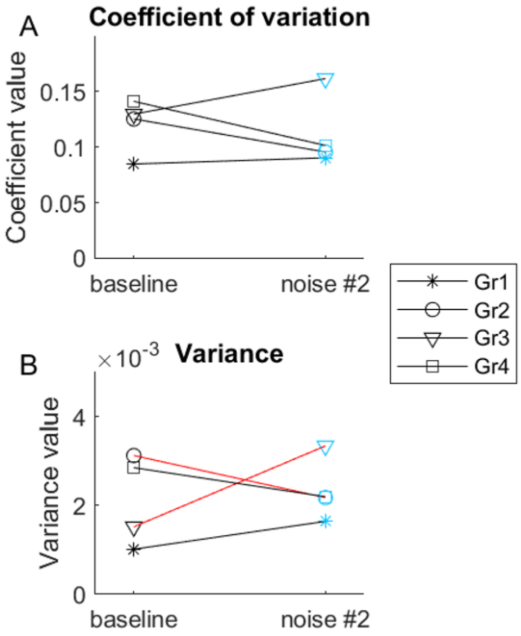

Supplementary Figure 5: Measure of variability of the centroid

(A) plotted is the coefficient of variation of the normalized centroid for each group for the summarized baseline and noise #2 data sets. The coefficients are fairly stable after noise exposure and only Group 3 shows a slight increase. (B) shows the variance of the normalized centroid for the summarized baseline and noise #2 data sets (matched for each group for the minimum number of observation bins over both conditions). A two sample Bartlett test for each group judged whether the baseline and noise #2 variances are unequal (α=0.05 and n=2, $T > \chi^{\wedge 2}_{1-\alpha,n-1}=3.841$; T=2.952 for Group 1, T=6.041 for Group 2, T=13.396 for Group 3, T=0.838 for Group 4). Red lines between baseline and noise #2 variances indicate when the variances were judged as unequal for a given group.

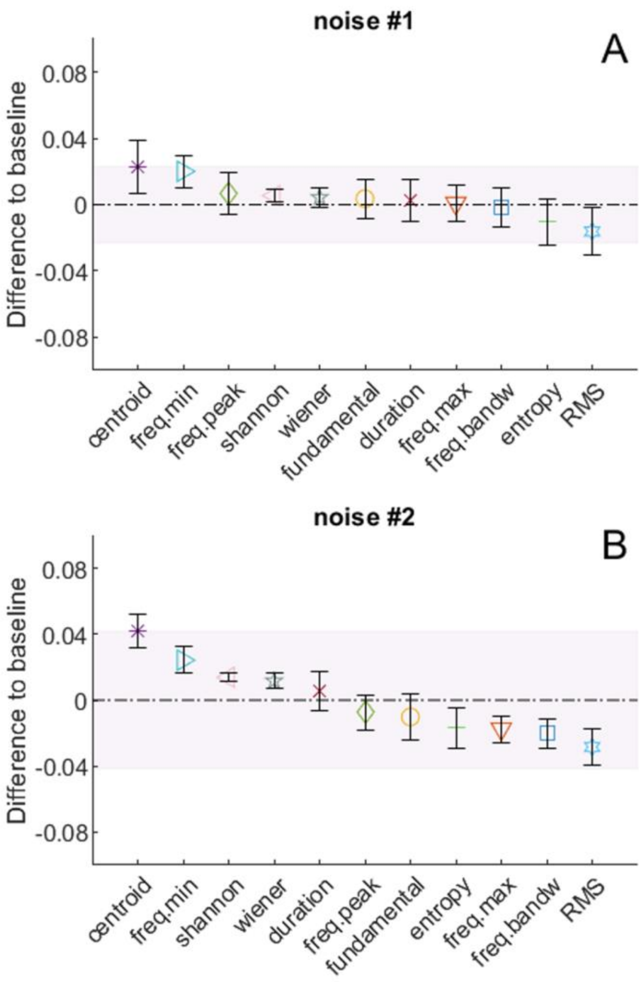

Supplementary Figure 6: Effect of noise on all acoustic parameters for Groups 1 and 2

(A) the mean shift compared to the summarized baseline data set for Groups 1 and 2 for the summarized noise #1 data set for all extracted acoustic parameters (values are normalized to allow comparison); plots indicate the differences of the least square mean and their 95% confidence intervals. The strongest absolute shift is seen for the centroid followed by minimum frequency (freq.min), RMS (root mean square) and entropy. Values and significance levels are shown in Table 1. (B) the mean shift compared to the summarized baseline data set for Groups 1 and 2 for the summarized noise #2 data set, where the strongest shift is again found for the centroid followed by RMS, minimum frequency and frequency bandwidth (freq.bandw). Values and significance levels are shown in Table 2. Both plots indicate an overall effect of noise on acoustic call parameters, but stronger effects are seen for the second noise condition. Peak frequency and maximum frequency are abbreviated with freq.peak and freq.max, respectively.

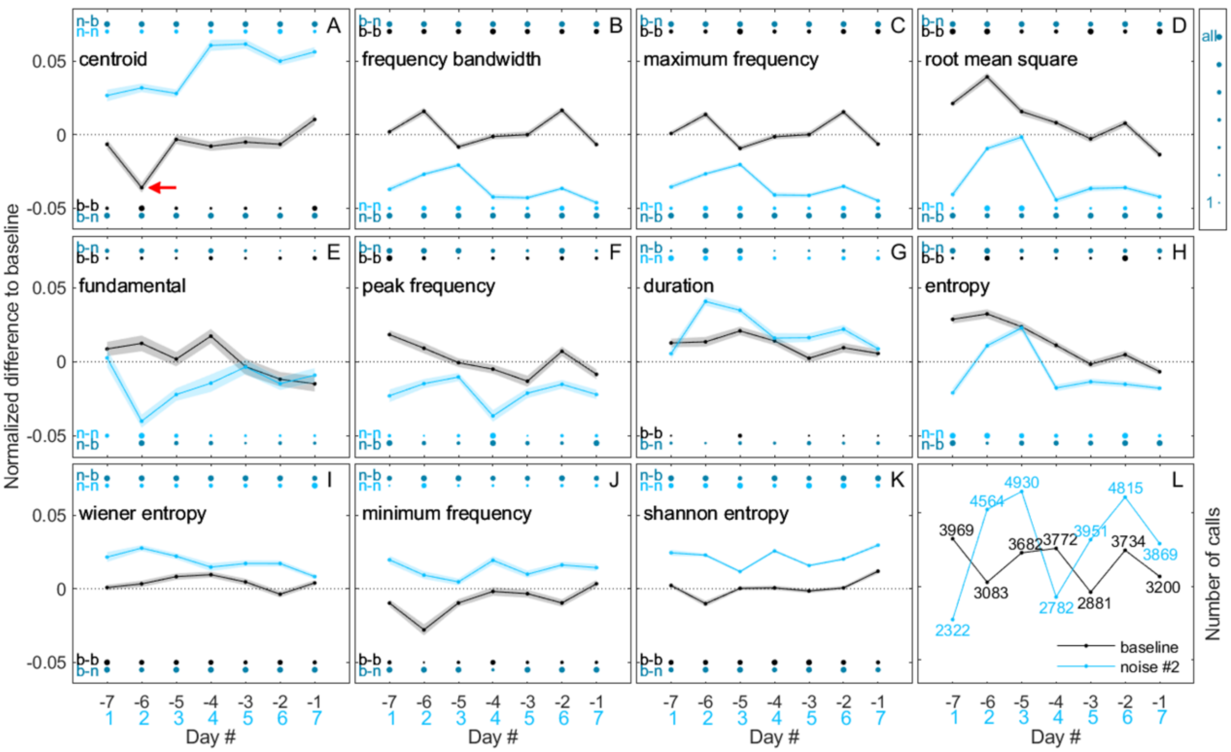

Supplementary Figure 7: Comparison of daily fluctuations

Plotted for each call parameter and across all groups is the normalized difference to the overall mean baseline on a day-by-day basis. The standard error is indicated by the shaded areas around each curve. Each data point is calculated by subtracting the mean value across all groups (for baseline or noise #2) from the overall mean baseline value for each day. Data is taken from the 1-week data sets (last week of baseline and first week of silence after noise #2). The row of dots ‘n-b’ depicts for each noise #2 data point how often it is significant to all baseline data points (range is from 0 – 7 days). The row of dots (n-n) depicts for each day how often a noise #2 data point is significant to the other noise #2 data points (range is from 0 – 6 days). The same calculation is done for the baseline data points (b-n and b-b). The size of the dot scales with increasing number of significant days (see legend to the right of D: ‘all’ corresponds to 7 days for the n-b and b-n rows and to 6 days for the n-n and b-b rows; Wilcoxon rank sum test). It is important to note, that the centroid values (A) only reach maximum significant days for the n-b and b-n comparisons except for the b-b data point marked by the red arrow. (L) shows how many calls went into each data point per day.

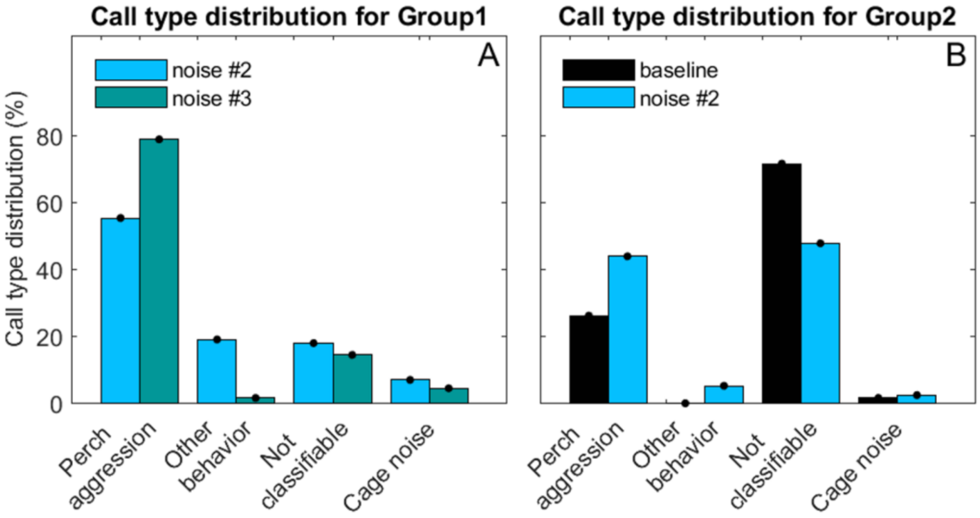

Supplementary Figure 8: Call type distribution for manual call classification

The results for the manual call classification based on behavior observed in the associated video frames are depicted for Group 1 (A) and for Group 2 (B). The dominant behavior found for both groups is 'Perch Aggression', but Group 1 (consisting of both males and females) exhibited a second frequent behavior of 'Mating Aggression' comprising 16% of the calls (included in 'Other behavior'). Due to the fact that bats live in very close proximity to each other which results in occlusions, a large percentage of calls could not be classified according to a behavior and were determined as 'Not Classifiable'. Possible contamination of observed results by cage noise was excluded as these only amounted to a very small percent of overall calls (6% for Group 1 and 2% for Group 2).

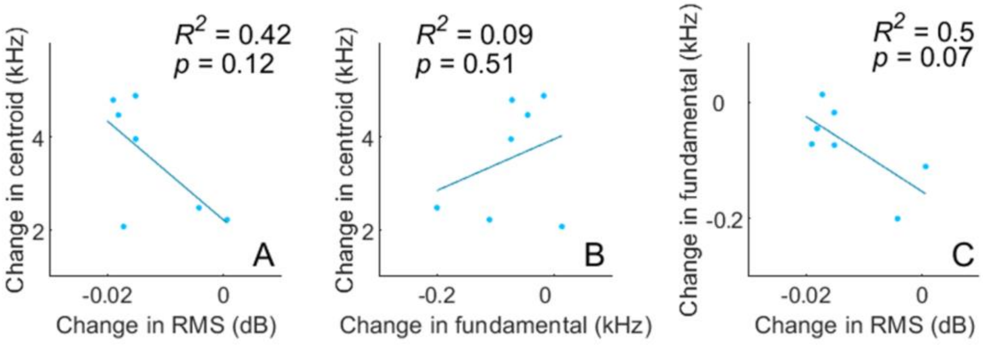

Supplementary Figure 9: Correlation between noise-induced changes in three call parameters

Correlation between changes in spectral centroid and RMS (root mean square = call amplitude). (A) spectral centroid, (B) fundamental and (C) fundamental and RMS. Data points for the correlation were taken from Supplementary Figure 7. These points reflect the mean changes in call parameters after noise #2 relative to the baseline for each day of the week in silence and were calculated across all groups. Thus, there were 7 data points in total. None of the correlations between the parameters is significant (Pearson's correlation using t-test).

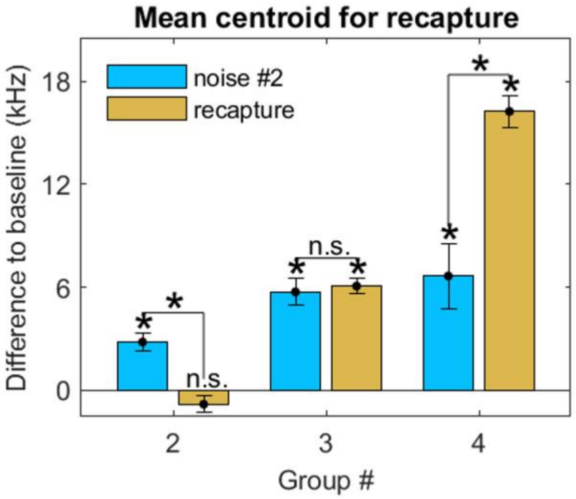

Supplementary Figure 10: Uncontrolled group effect on vocalizations

After the week(s) of silence which followed exposure to the second noise condition, we released Groups 2, 3 and 4 back to bat colonies which are comprised of hundreds of bats. After a period of one month, each group was recaptured and again recorded in silence for the duration of one month. Overall, we recorded 31387, 37424 and 14780 calls for Groups 2, 3 and 4, respectively. Data underwent the summarized process as described in Methods. Plotted is the mean centroid and the corrected standard error in terms of its difference to baseline. Interestingly, only Group 2 exhibited a downward shift of its centroid close to the original baseline of the group. This shift of -0.835 ± 0.474 kHz to baseline was not significant (z=1.763 and p=0.078, n=692 bins, z-test through AR(1) model), but the shift of -3.621 ± 0.382 kHz to noise #2 is significant (z=9.485 and p<0.001, n=709 bins, z-test through AR(1) model). The mean centroids of Groups 3 and 4 remained elevated compared to baseline and had therefore not shifted back to their original baseline; the shifts were 6.065 ± 0.435 kHz and 16.258 ± 0.947 kHz, respectively, and were significant compared to baseline (z=13.93 and p<0.001, n=736 bins for Group 3, z=17.17 and p<0.001, n=357 bins for Group 4, z-test through AR(1) model). For Group 3, the difference to noise #2 was -0.032 ± 0.33 kHz and not significant (z=0.097 and p=0.923, n=2092 bins, z-test through AR(1) model), and for Group 4 it was 11.177 ± 0.553 kHz and significant (z=20.218 and p<0.001, n=442 bins, z-test through AR(1) model). Notations above each bar indicate significance (*) or not (n.s.) when compared to baseline or the noise condition, with α=0.0045. One difference between Groups 2, 3

and 4, was the colony structure they were released back to. Groups 3 and 4 were released to a recently established male-only colony. Group 2, on the other hand, was released and then recaptured from its original mixed-gender colony. Group 2, therefore, was potentially exposed to a different colony environment than Groups 3 and 4. It is also possible, that only Groups 3 and 4 (when reintroduced to the same group after recapture) applied their group signature calls associated with this group (see second to last paragraph in the main text). Another explanation might be found in theories concerning motor learning. Context-dependent motor memory consolidation has been studied in motor learning and interference studies for humans, primates and birds[1,2,3,4,5,6,7], and it has been shown that the environmental conditions in which a task is performed and learned, contains information that is then associated with a task and facilitates memory retrieval[8,9,10,11,12,13]. Furthermore, long-term retention underlying such motor memories seems to be more dependent on the amount of times an action has been practiced and not on how well the action is performed[4,5]. Groups 3 and 4 had remained for a much longer duration (5-11 weeks) in the experimental setup than Group 2 (1 week) after the noise playback had ceased. These two groups might have 'learned' these modified calls in a specific context, in this case the sound box. But as we had neither knowledge concerning the social status of these bats in the colony nor of the vocal interactions the bats were exposed to or were engaged in during this time period, we can only speculate. Further studies are needed to bring definite insight into these hypotheses.

Supplementary Tables:

noise #1	centroid (kHz)	duration (ms)	RMS (dB)	entropy (dB)	peak frequency (kHz)	frequency bandwidth (kHz)	minimum frequency (kHz)	maximum frequency (kHz)	fundamental (kHz)	Shannon entropy (dB)	Wiener entropy (dB)
Group1	0.471	-0.225	-0.181	-0.177	1.308*	0.937	0.303*	1.237	-0.038	-0.35	-0.156
Group2	1.69	-1.601	-0.729	-1.124	-0.064	-0.204	0.196*	-0.008	0.112	-1.227	0.064

Table 1: Acoustic parameters noise #1

Shown are the acoustic parameters for summarized noise #1 data sets for Group 1 and 2, asterisks indicate significance from summarized baseline data sets (z-test through AR(1) model, at 5% significance level and corrected for multiple testing with Bonferroni for 11 parameters). Reported are the differences of least squares means.

noise #2	centroid (kHz)	duration (ms)	RMS (dB)	entropy (dB)	peak frequency (kHz)	frequency bandwidth (kHz)	minimum frequency (kHz)	maximum frequency (kHz)	fundamental (kHz)	Shannon entropy (dB)	Wiener entropy (dB)
Group1	3.118*	11.08*	-0.395	1.558	-0.99	-2.448*	0.141	-2.305*	-0.298*	-3.31*	0.41*
Group2	2.795*	-3.671	-1.202*	-1.882*	0.009	-1.541*	0.317*	-1.225	0.098	-2.539*	0.064
Group3	5.724*	4.088*	-3.22*	-4.716*	-0.916*	-4.88*	-0.226*	-5.104*	-0.142*	-7.4*	2.227*
Group4	6.65*	3.128	-2.732*	-4.708*	-1.894	-4.173*	0.028	-4.14*	-0.146	-8.72*	1.416*

Table 2: Acoustic parameters noise #2

Shown are the acoustic parameters for summarized noise #2 data sets for all Groups, asterisks indicate significance from summarized baseline data sets (z-test through AR(1) model, at 5% significance level and corrected for multiple testing with Bonferroni for 11 parameters). Reported are the differences of least squares means.

noise #3	centroid (kHz)	duration (ms)	RMS (dB)	entropy (dB)	peak frequency (kHz)	frequency bandwidth (kHz)	minimum frequency (kHz)	maximum frequency (kHz)	fundamental (kHz)	Shannon entropy (dB)	Wiener entropy (dB)
Group1	2.883*	11.722*	-0.436	1.027	-2.436*	-1.598*	0.036	-1.557*	-0.587*	-2.34*	0.208

Table 3: Acoustic parameters noise #3

Shown are the acoustic parameters for the summarized noise #3 data set for Group 1, asterisks indicate significance from the summarized baseline data set (z-test through AR(1) model, at 5% significance level and corrected for multiple testing with Bonferroni for 11 parameters). Reported are the differences of least squares means.

noise #2 all groups	centroid (kHz)	duration (ms)	RMS (dB)	entropy (dB)	peak frequency (kHz)	frequency bandwidth (kHz)	minimum frequency (kHz)	maximum frequency (kHz)	fundamental (kHz)	Shannon entropy (dB)	Wiener entropy (dB)
1 week	3.563*	1.728	-1.377*	-1.623*	-0.526*	-2.94*	0.133*	-2.81*	-0.055	-4.89*	0.276*
5000 calls	2.997*	5.871*	-0.636	0.538	-0.899*	-3.523*	0.008	-3.511*	-0.124*	-4.667*	0.429*

Table 4: Acoustic parameters for noise #2

Shown are the acoustic parameters for noise #2 across all groups for the summarized 1-week and summarized 5000-data set, asterisks indicate significance from baseline (t-test though mixed linear model, at 5% significance level and corrected for multiple testing with Bonferroni for 11 parameters). Reported are the differences of least squares means.

Group	Mean (n^a) baseline (kHz)	Mean (n^a) noise #2 (kHz)	Pooled SD (kHz)	*r1*
1	0.012 (53)	3.449 (81)	2.839	0.144
2	0.007 (188)	3.307 (261)	4.068	0.264
3	0.017 (106)	4.939 (90)	3.945	0.533
4	-0.051 (72)	2.627 (51)	4.506	0.506

Table 5: Generalized least squares estimates for 1-week data set

Shown are the generalized least squares estimates for baseline and noise #2, the pooled standard deviation (SD) and the 1st order autocorrelation (r_1) for each group for the summarized 1-week data sets. [a] every 70 calls were averaged to one observation bin; n is the number of observation bins. The total number of calls is ~ 70xn.

Group	Mean (n^a) baseline (kHz)	Mean (n^a) noise #2 (kHz)	Pooled SD (kHz)	*r1*
1	0.004 (71)	3.222 (71)	3	0.208
2	-0.018 (71)	2.86 (71)	4.167	0.278
3	-0.01 (71)	3.486 (71)	3.835	0.49
4	-0.083 (71)	2.333 (71)	4.443	0.457

Table 6: Generalized least squares estimates for 5000-data set

Shown are the generalized least squares estimates for baseline and noise #2, the pooled standard deviation (SD) and the 1st order autocorrelation (r_1) for each group for the summarized 5000-data sets. [a] every 70 calls were averaged to one observation bin; n is the number of observation bins. The total number of calls is ~ 70xn.

Coefficient of variation		centroid	duration	RMS	entropy	peak frequency	frequency bandwidth	minimum frequency	maximum frequency	fundamental	Shannon entropy	Wiener entropy
baseline	Group1	0.085	0.166	0.093	0.288	0.07	0.05	0.124	0.044	0.085	-0.205	0.048
	Group2	0.125	0.168	0.198	0.373	0.076	0.109	0.164	0.094	0.116	-0.397	0.039
	Group3	0.13	0.1	0.165	0.288	0.057	0.033	0.076	0.03	0.089	-0.533	0.439
	Group4	0.141	0.117	0.256	0.45	0.148	0.06	0.053	0.055	0.138	-0.93	0.273
noise #2	Group1	0.09	0.228	0.127	0.378	0.105	0.051	0.157	0.044	0.155	-0.255	0.047
	Group2	0.096	0.146	0.148	0.305	0.076	0.069	0.106	0.059	0.134	-0.338	0.039
	Group3	0.162	0.107	0.246	0.4	0.066	0.052	0.08	0.048	0.077	-.0.725	0.43
	Group4	0.102	0.097	0.119	0.265	0.077	0.072	0.042	0.066	0.106	-0.585	0.262

Table 7: Coefficient of variation

Shown is the coefficient of variation for each acoustic parameter for the baseline and noise #2 for each group for the summarized 1-week data set.

Variance		centroid	duration	RMS	entropy	peak frequency	frequency bandwidth	minimum frequency	maximum frequency	fundamental	Shannon entropy	Wiener entropy
baseline	Group1	0.001	0.001	0.001	0.001	0.001	0.001	0.001	0.001	0.002	0	0
	Group2	0.003	0.003	0.003	0.003	0.002	0.002	0.002	0.002	0.003	0	0.001
	Group3	0.002	0.001	0.001	0	0.001	0	0	0	0.003	0	0
	Group4	0.003	0.001	0.001	0	0.003	0.001	0	0.001	0.003	0	0
noise #2	Group1	0.002	0.003	0.001	0.001	0.003	0.001	0.002	0.001	0.007	0	0
	Group2	0.002	0.003	0.002	0.002	0.002	0.001	0.001	0.001	0.004	0	0.001
	Group3	0.003	0.001	0.001	0	0.001	0.001	0	0.001	0.002	0	0
	Group4	0.002	0.001	0	0	0.001	0.001	0	0.001	0.003	0	0

Table 8: Variance

Shown is the normalized variance of each acoustic parameter for baseline and noise #2 for each group for the summarized 1-week data set.

noise #2 all groups	peak-to-peak (dB)
1 week	-1.001*
5000 calls	-0.671*

Table 9: Peak-to-peak amplitude

Shown is the peak-to-peak amplitude for noise #2 across all groups for the summarized 1-week and summarized 5000-data set, asterisks indicate significance from baseline (z-test through AR(1) model, at 5% significance level and corrected for multiple testing with Bonferroni for 11 parameters). Reported are the differences of least squares means.

Supplementary References:

1. Brashers-Krug, T., Shadmehr, R. & Bizzi, E. Consolidation in human motor memory. *Nature* **382,** 252–255 (1996).

2. Shadmehr, R. & Brashers-Krug, T. Functional stages in the formation of human long-term motor memory. *J. Neurosci.* **17,** 409–19 (1997).

3. Bock, O., Schneider, S. & Bloomberg, J. Conditions for interference versus facilitation during sequential sensorimotor adaptation. *Exp. Brain Res.* **138,** 359–365 (2001).

4. Fernández-Ruiz, J. & Díaz, R. Prism adaptation and aftereffect: Specifying the properties of a procedural memory system. *Learn. Mem.* **6,** 47–53 (1999).

5. Yin, P. B. & Kitazawa, S. Long-lasting aftereffects of prism adaptation in the monkey. *Exp. Brain Res.* **141,** 250–253 (2001).

6. Joiner, W. M. & Smith, M. A. Long-Term Retention Explained by a Model of Short-Term Learning in the Adaptive Control of Reaching. *J. Neurophysiol.* **100,** 2948–2955 (2008).

7. Tian, L. Y. & Brainard, M. S. Discrete Circuits Support Generalized versus Context-Specific Vocal Learning in the Songbird. *Neuron* **96,** 1168–1177.e5 (2017).

8. Wright, D. L. & Shea, C. H. Contextual dependencies in motor skills. *Mem. Cognit.* **19,** 361–370 (1991).

9. Chun, M. M. & Jiang, Y. Contextual Cueing: Implicit Learning and Memory of Visual Context Guides Spatial Attention. *Cogn. Psychol.* **36,** 28–71 (1998).

10. Smith, S. M. & Vela, E. Environmental context-dependent memory: A review and meta-analysis. *Psychon. Bull. Rev.* **8,** 203–220 (2001).

11. Lee, Y. Y., Winstein, C. J. & Fisher, B. E. Role of the dorsolateral prefrontal cortex in context-dependent motor performance. *Eur. J. Neurosci.* **43,** 954–960 (2016).

12. Lee, Y. Y. & Fisher, B. E. The Effect of Practice Schedule on Context-Dependent Learning. *Journal of Motor Behavior* 1–8 (2018). doi:10.1080/00222895.2018.1437020

13. Rochet-Capellan, A. & Ostry, D. J. Simultaneous Acquisition of Multiple Auditory-Motor Transformations in Speech. *J. Neurosci.* **31,** 2657–2662 (2011).
